# PTG‐Dependent Glycogen Metabolic Dysfunction Drives Impaired Adipose Browning: A Novel Mechanism Linking PM_2.5_ to Metabolic Disorders

**DOI:** 10.1002/advs.202512589

**Published:** 2026-01-09

**Authors:** Limin Wang, Renjie Hu, Yanxi Chai, Ping He, Sanduo Li, Lisha Zhao, Wenbin Zhao, Lu Zhang, Li Qin, Ran Li, Xiaoli Hou, Qinghua Sun, Cuiqing Liu

**Affiliations:** ^1^ School of Public Health Zhejiang Chinese Medical University Hangzhou Zhejiang China; ^2^ Zhejiang International Science and Technology Cooperation Base of Air Pollution and Health Hangzhou Zhejiang China; ^3^ Academy of Chinese Medical Sciences Zhejiang Chinese Medical University Hangzhou Zhejiang China

**Keywords:** β3‐adrenergic receptor, fine particulate matter, glycogen metabolism, iWAT browning, protein targeting to glycogen, vascular endothelial growth factor B

## Abstract

Fine particulate matter (PM_2.5_) contributes to metabolic dysfunction, but its effects on adipose tissue browning remain unclear. Here, we showed that PM_2.5_ exposure inhibited inguinal white adipose tissue (iWAT) browning by downregulating protein targeting to glycogen (PTG), disrupting glycogen homeostasis. PTG overexpression in iWAT restored glycogen metabolism, thermogenesis, and mitochondrial function, reversing PM_2.5_‐induced impairment in iWAT browning and metabolic disorders. Mechanistically, PTG negatively regulated vascular endothelial growth factor B (VEGFB), and VEGFB knockdown rescued browning. Activation of β3‐adrenergic receptor (ADRB3) mitigated PM_2.5_’s effects by restoring PTG and normalizing VEGFB, defining the ADRB3‐PTG‐VEGFB axis as central to PM_2.5_‐induced metabolic dysfunction. Our findings identify adipose glycogen metabolism as a target for countering environmental metabolic disruption.

## Graphical abstract

This study provides the first evidence that PM_2.5_ impairs iWAT browning via PTG‐mediated glycogen metabolism disruption, which is initiated by ADRB3 inhibition and subsequently triggers VEGFB upregulation. It thereby delineates the ADRB3‐PTG‐VEGFB axis as central to PM_2.5_‐induced metabolic dysfunction and identifies adipose glycogen metabolism as a novel therapeutic target against environmental metabolic disruption.

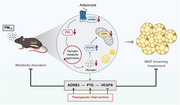

## 1 Introduction

Fine particulate matter (PM_2.5_), defined as particles with aerodynamic diameters ≤ 2.5 µm, represents a critical environmental pollutant containing toxic components derived from vehicular emissions and industrial processes [1, 2, 3, 4]. Extensive evidence has established that PM_2.5_ exposure increased susceptibility to metabolic syndrome, a disorder characterized by central obesity [5, 6, 7], glucose metabolism disruption [8, 9, 10], and dyslipidemia [11, 12]. As the body's largest endocrine organ, adipose tissue, comprising white, beige, and brown adipocytes, plays a fundamental role in metabolic regulation [13, 14, 15]. Notably, inguinal white adipose tissue (iWAT) acquires brown adipose tissue (BAT)‐like thermogenic capacity through cold exposure or β‐adrenergic receptor‐stimulated browning, which enhances energy expenditure via uncoupling protein 1 (UCP1)‐dependent thermogenesis to meet both cold‐adaptive thermoregulatory demands and metabolic syndrome protection [16, 17, 18]. Studies from Lobato et al. [19] and our group [20] demonstrated that PM_2.5_ exposure significantly impaired white adipose tissue (especially iWAT) browning, yet the precise molecular mechanism remains poorly understood, representing a critical knowledge gap in environmental metabolic research.

Glycogen, the primary glucose storage polymer in mammals, serves as a vital energy reservoir [21, 22]. Toxicological evidence revealed that PM_2.5_ exposure reduced hepatic glycogen stores [23] and suppressed hepatic glycogen synthesis [24]. While adipose tissues typically maintain lower basal glycogen content compared to liver or muscle, they demonstrated dynamic glycogen fluctuations during fasting‐refeeding cycles, suggesting their involvement in acute metabolic regulation [25]. Notably, the impact of PM_2.5_ on iWAT glycogen metabolism remains unexplored. Protein targeting to glycogen (PTG), encoded by *Ppp1r3c* and highly expressed in adipocytes [26], serves as a critical regulatory subunit of protein phosphatase‐1 (PP1) that orchestrates glycogen synthesis [27]. Recent studies revealed that adipocyte‐specific PTG deletion reduced beige adipocyte glycogen content, impairing UCP1 expression and blunting cold‐ or β‐adrenergic receptor‐stimulated weight loss in obese mice [28]. However, PTG's potential role in PM_2.5_‐mediated iWAT browning impairment has not been investigated.

β3‐adrenergic receptor (ADRB3) serves as the pivotal mediator of adaptive thermogenesis within both brown and beige adipocytes [29, 30]. Our prior work identified ADRB3 methylation and subsequent downregulation as a key mechanism in PM_2.5_‐impaired iWAT browning [20]. Supporting this connection, studies by Markan et al. demonstrated that β‐adrenergic signaling enhanced glycogen turnover in PTG transgenic mouse models [31], suggesting the crosstalk between β‐adrenergic signaling and glycogen metabolism. Nevertheless, whether ADRB3 mediates PM_2.5_‐impaired iWAT browning via PTG‐regulated glycogen metabolism remains unknown.

In this study, we employed a whole‐body inhalation exposure system to investigate PM_2.5_’s effects on iWAT browning and glycogen metabolism. Through integrated in vivo and in vitro approaches, we elucidated PTG's central role in mediating PM_2.5_‐suppressed iWAT browning. The database predictions and subsequent validation supported a novel regulatory axis involving the ADRB3‐PTG‐VEGFB signaling pathway. These findings provided mechanistic insights into PM_2.5_‐inhibited iWAT browning and identified potential therapeutic targets for environment‐related metabolic disorders.

## 2 Results

### 2.1 PM_2.5_ Characteristics

**FIGURE 1:**
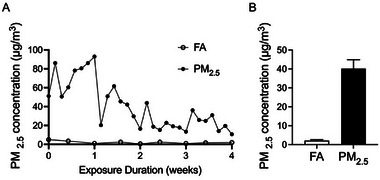
Concentrations of PM_2.5_ at the study site. (A) Daily PM_2.5_ concentrations in the FA and PM_2.5_ chamber in the area where the exposure system was located. (B) Average PM_2.5_ concentrations in the FA and PM_2.5_ chamber during the exposure period. Data were presented as mean ± SEM.

The whole‐body inhalation exposure system employed in this study represents a validated method for PM_2.5_ exposure modeling [32, 33]. This system directly samples ambient outdoor air, with daily PM_2.5_ exposure concentrations dynamically reflecting fluctuations in outdoor air quality, thereby accurately recapitulating real‐world human exposure patterns and scenarios. The concentration curves of PM_2.5_ during the exposure period are shown in Figure 1A. In the FA chamber and the PM_2.5_ chamber, the mice were exposed to average daily PM_2.5_ concentrations of 2.15 ± 0.47 µg/m^3^ and 40.18 ± 4.70 µg/m^3^, respectively (Figure 1B). Table 1 summarizes PM_2.5_ composition during exposure, with sulfate (SO_4_^2−^), aluminum (Al), and arsenic (As) being the most abundant species in water‐soluble inorganic ions, metallic elements, and non‐metallic elements, respectively.

**TABLE 1:** Main composition of PM_2.5_ in the PM_2.5_ chamber during the exposure period.

Element	Mean	Standard deviation	Minimum	Median	Maximum
Water‐soluble inorganic ions (µg/m^3^)					
Cl^−^	0.69	1.71	0.02	0.02	4.57
SO_4_^2−^	2.97	4.10	0.10	0.63	9.14
NO_3_^−^	2.61	1.11	1.48	2.20	4.26
NH_4_^+^	1.30	1.35	0.05	0.88	4.16
Metallic elements (ng/m^3^)					
Al	188.10	415.50	20.90	28.20	1130
Mn	21.96	7.92	7.24	21.90	30.80
Ni	4.93	10.42	0.41	0.74	28.50
Cd	2.76	3.37	0.33	1.30	9.80
Sb	1.95	1.55	0.97	1.47	5.40
Hg	0.08	0.11	0.04	0.04	0.32
Non‐metallic elements (ng/m^3^)					
As	3.04	2.03	1.61	2.51	7.43
Se	2.07	1.05	0.83	1.83	3.6

### 2.2 PM_2.5_ Exposure Inhibited Systemic Metabolism and iWAT Browning

**FIGURE 2:**
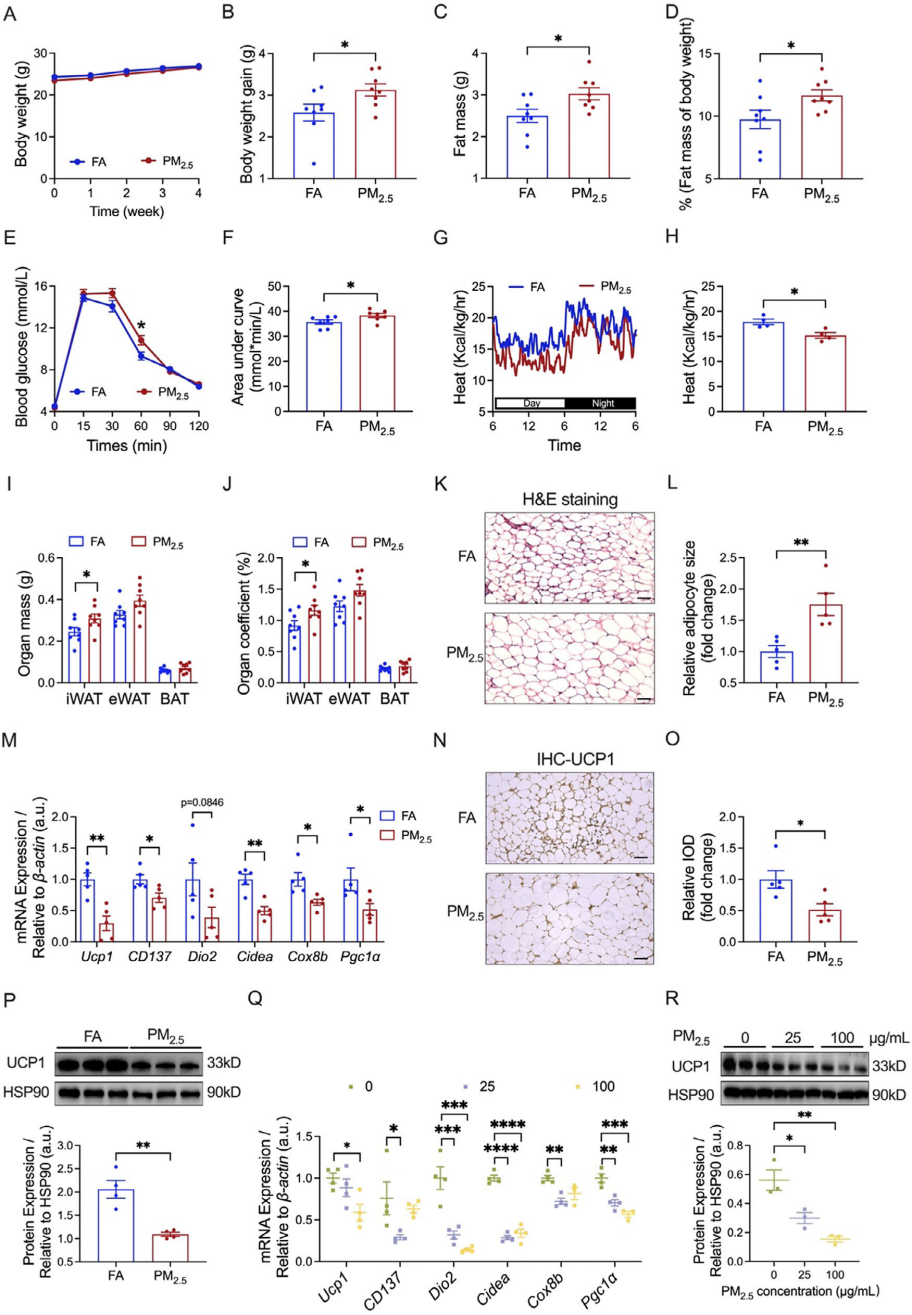
Effects of PM_2.5_ exposure on systemic metabolism and iWAT browning. (A,B) Body weight (A) and body weight gain (B) of mice (*n* = 8 per group). (C,D) Fat mass (C) and the ratio of fat mass to body weight (D) of mice (*n* = 8 per group). (E,F) GTT (E) and AUC of GTT (F) of mice (*n* = 7 per group). (G,H) Heat production of mice (*n* = 4 per group). Line graph (G) indicates variation patterns of heat production of mice over a 24 h period, column graph (H) indicates the mean values of heat production of mice over a 24 h period. (I,J) Adipose mass (I) and adipose coefficient (J) of mice (*n* = 8 per group). (K,L) Representative images (K) and quantitative analysis (L) of H&E staining in iWAT sections (*n* = 5 per group); Scale bar = 50 µm. (M) mRNA expression of thermogenic genes in iWAT (*n* = 5 per group). (N,O) Representative images (N) and relative IOD (O) of immunohistochemistry in iWAT sections stained with UCP1 antibody (*n* = 5 per group); Scale bar = 50 µm. (P) Representative bands (top) and quantitative analysis (bottom) of UCP1 in iWAT (*n* = 4 per group). (Q) mRNA expression of thermogenic genes in 3T3‐L1 adipocytes (*n* = 4 per group). (R) Representative bands (top) and quantitative analysis (bottom) of UCP1 in 3T3‐L1 adipocytes (*n* = 3 per group). Data were presented as mean ± SEM, analyzed by unpaired, two‐tailed Student's *t*‐test (B‐D, F, H‐J, L‐P), one‐way ANOVA with Tukey's test (Q,R), or two‐way ANOVA with Tukey's multiple comparisons test (E). ^*^ *p* < 0.05, ^**^ *p* < 0.01, ^***^ *p* < 0.001, ^****^ *p* < 0.0001.

First, we investigated the impacts of PM_2.5_ exposure on whole‐body metabolism. As depicted in Figure 2A, although PM_2.5_ exposure did not notably influence the body weight of the mice, it significantly increased body weight gain compared to the control group (Figure 2B). PM_2.5_‐exposed mice also showed significant increases in fat mass (Figure 2C) and its ratio to body weight (Figure 2D). While the change in lean mass was not statistically significant (Figure S1A), the ratio of lean mass to body weight in the PM_2.5_‐exposed mice was lower than that in the FA‐exposed mice (Figure S1B). An intraperitoneal glucose tolerance test (IPGTT) was performed to investigate the effect of PM_2.5_ exposure on glucose homeostasis. Figure 2E,F reveals significantly higher blood glucose levels at 60 min after glucose administration and area under the curve (AUC) in mice exposed to PM_2.5_ exposure, whereas a significant difference in blood glucose was only noticed at 120 min after insulin administration between FA and PM_2.5_ groups in the insulin tolerance test (ITT) (Figure S1C,D). Oxygen consumption (*p* = 0.0704) (Figure S1E,F) and carbon dioxide release (*p* = 0.0538) (Figure S1G,H) tended to decrease after PM_2.5_ exposure, while heat production was significantly inhibited (Figure 2G,H). These findings confirmed that PM_2.5_ exposure caused metabolic disorders in mice.

Subsequently, we delved into the impact of PM_2.5_ exposure on the browning of iWAT. PM_2.5_ exposure significantly increased both the mass (Figure 2I) and organ coefficient (Figure 2J) of iWAT, with no significant changes observed in the mass or organ coefficient of epididymal white adipose tissue (eWAT) or BAT (Figure 2I,J). Hematoxylin&Eosin (H&E) staining exhibited that PM_2.5_ exposure altered the morphology of iWAT, characterized by the increased size of adipocytes (Figure 2K,L). During the browning process, iWAT exhibited an upregulation of thermogenic genes [34], with UCP1 serving as a golden hallmark indicative of iWAT browning [35]. We found the mRNA expression of marker molecules linked to adipocyte thermogenesis and beige adipocytes, such as *Ucp1*, tumor necrosis factor receptor superfamily 9 (*CD137*), cell death‐inducing DNA fragmentation factor alpha‐like effector A (*Cidea*), cytochrome c oxidase subunit 8B (*Cox8b*), and peroxisome proliferator‐activated receptor gamma, coactivator 1 alpha (*Pgc1α*), was highly downregulated in iWAT of PM_2.5_ exposed‐mice (Figure 2M). Both immunohistochemistry (Figure 2N,O) and western blotting (Figure 2P) analysis revealed a significant reduction in PM_2.5_‐induced UCP1 expression in iWAT of mice. Additionally, we explored the influence of different concentrations of PM_2.5_ on the iWAT browning at the cellular level. As shown in Figure 2Q, PM_2.5_ at 25 µg/mL significantly suppressed the gene expression levels of *CD137*, deiodinase iodothyronine, type II (*Dio2*), *Cidea*, *Cox8b*, and *Pgc1α* in 3T3‐L1 adipocytes, a classic cell model of the adipocyte. When treated with 100 µg/mL PM_2.5_, there was a significant decline in the mRNA expression levels of *Ucp1*, *Dio2*, *Cidea*, and *Pgc1α* in 3T3‐L1 adipocytes (Figure 2Q). Western blotting analysis further demonstrated that PM_2.5_ at different concentrations also led to a marked reduction in the protein expression of UCP1 (Figure 2R). These findings suggested that PM_2.5_ exposure inhibited iWAT browning.

### 2.3 PM_2.5_ Exposure Suppressed Glycogen Metabolism in iWAT

**FIGURE 3:**
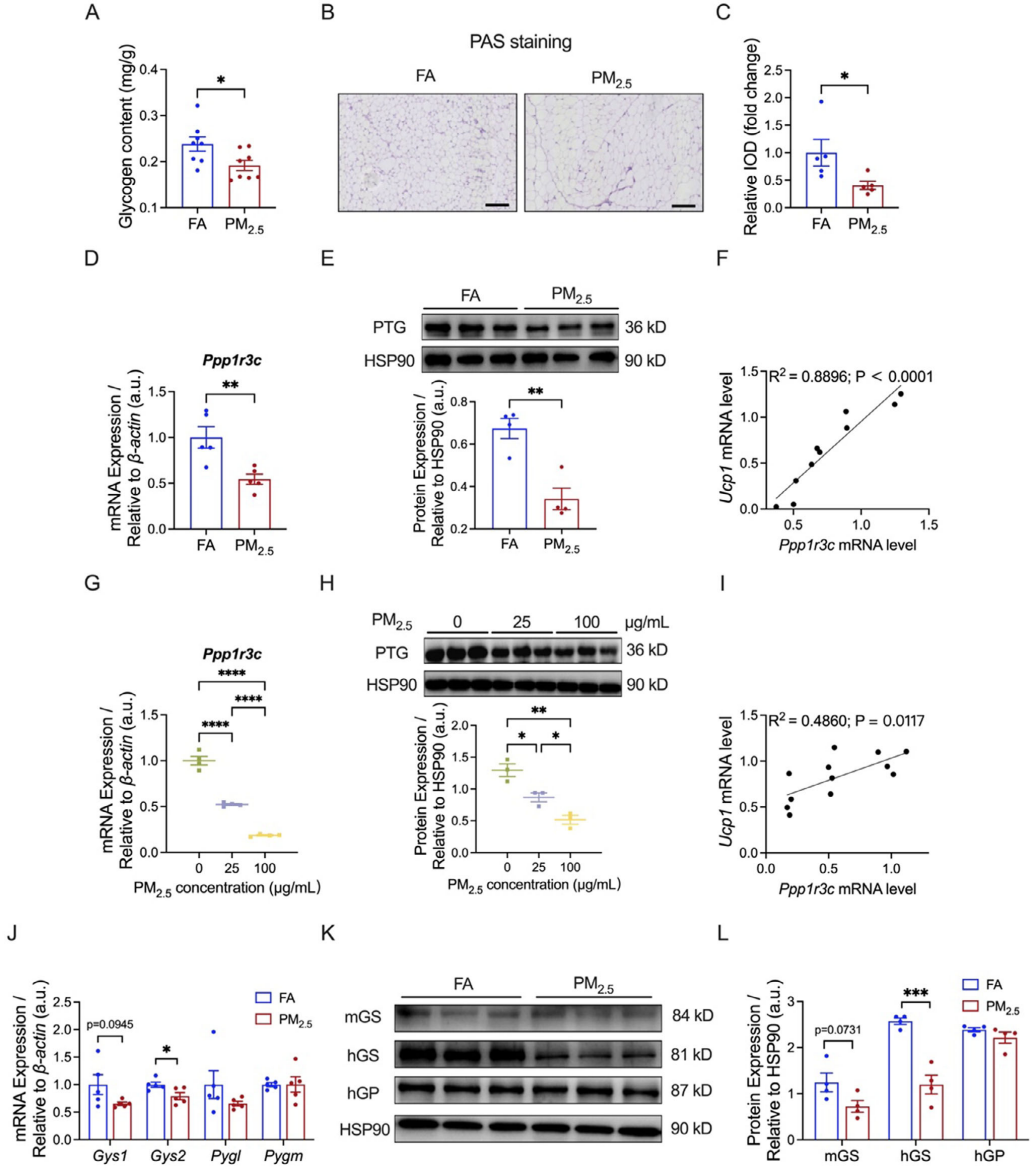
Effects of PM_2.5_ exposure on glycogen metabolism in iWAT. (A) Glycogen content in iWAT of mice (*n* = 8 per group). (B,C) Representative images (B) and relative IOD (C) of PAS staining in iWAT sections (*n* = 5 per group); Scale bar = 50 µm. (D) mRNA expression of *Ppp1r3c* in iWAT (*n* = 5 per group). (E) Representative bands (top) and quantitative analysis (bottom) of PTG in iWAT (*n* = 4 per group). (F) Correlation of *Ucp1* mRNA levels in iWAT with *Ppp1r3c* mRNA levels (*n* = 10). (G) mRNA expression of *Ppp1r3c* in 3T3‐L1 adipocytes (*n* = 4 per group). (H) Representative bands (top) and quantitative analysis (bottom) of PTG in 3T3‐L1 adipocytes (*n* = 3 per group). (I) Correlation of *Ucp1* mRNA levels in 3T3‐L1 adipocytes with *Ppp1r3c* mRNA levels (*n* = 12). (J) mRNA expression of rate‐limiting enzymes of glycogen metabolism in iWAT (*n* = 5 per group). (K,L) Representative bands (K) and quantitative analysis (L) of rate‐limiting enzymes of glycogen metabolism in iWAT (*n* = 4 per group). Data were presented as mean ± SEM, analyzed by unpaired, two‐tailed Student's *t*‐test (A–E, J, L) or one‐way ANOVA with Tukey's test (G, H). Correlations were examined with the non‐parametric Spearman correlation test (F, I). ^*^ *p* < 0.05, ^**^ *p* < 0.01, ^***^ *p* < 0.001, ^****^ *p* < 0.0001.

To elucidate the impact of PM_2.5_ exposure on glycogen metabolism of iWAT, we assessed glycogen content in iWAT and observed a significant reduction in glycogen levels subsequent to PM_2.5_ exposure (Figure 3A). In corroboration with this, Periodic Acid‐Schiff (PAS) staining revealed a decreased accumulation of glycogen after PM_2.5_ exposure (Figure 3B,C).

Since PTG serves as a critical scaffold protein that coordinates glycogen metabolism, we investigated its expression in iWAT of mice and 3T3‐L1 adipocytes following PM_2.5_ exposure. PM_2.5_ exposure led to a significant reduction in the mRNA expression level of *Ppp1r3c* and protein expression level of PTG (coded by *Ppp1r3c*) in iWAT of mice (Figure 3D,E). We conducted an analysis of the correlation between the mRNA expression of *Ppp1r3c* and that of *Ucp1* in iWAT of mice, and a significant positive correlation was observed (Figure 3F). In line with these findings, PM_2.5_ exposure also significantly decreased the mRNA level of *Ppp1r3c* and protein level of PTG in 3T3‐L1 adipocytes in a concentration‐dependent manner (Figure 3G,H). Moreover, a positive correlation was also observed between the mRNA expression levels of *Ppp1r3c* and *Ucp1* in 3T3‐L1 adipocytes (Figure 3I).

Given PTG's central role in glycogen metabolism, we further examined the expression levels of rate‐limiting enzymes involved in glycogen synthesis and breakdown in iWAT of mice. PM_2.5_ exposure significantly reduced the mRNA and protein level of hepatic glycogen synthase (hGS, coded by *Gys2*), with decreasing trends observed for the mRNA and protein level of muscle glycogen synthase (mGS, coded by *Gys1*) (Figure 3J‐L). In contrast, the expression levels of glycogen breakdown enzymes remained unchanged (Figure 3J‐L), suggesting downregulation of glycogen synthesis enzymes in iWAT following PM_2.5_ exposure.

These results suggested that reduced PTG expression might contribute to the PM_2.5_‐inhibited glycogen synthesis, which ultimately led to the reduction of glycogen content in iWAT.

### 2.4 PTG Overexpression Ameliorated PM_2.5_‐Induced Glycogen Metabolism Dysregulation in iWAT

**FIGURE 4:**
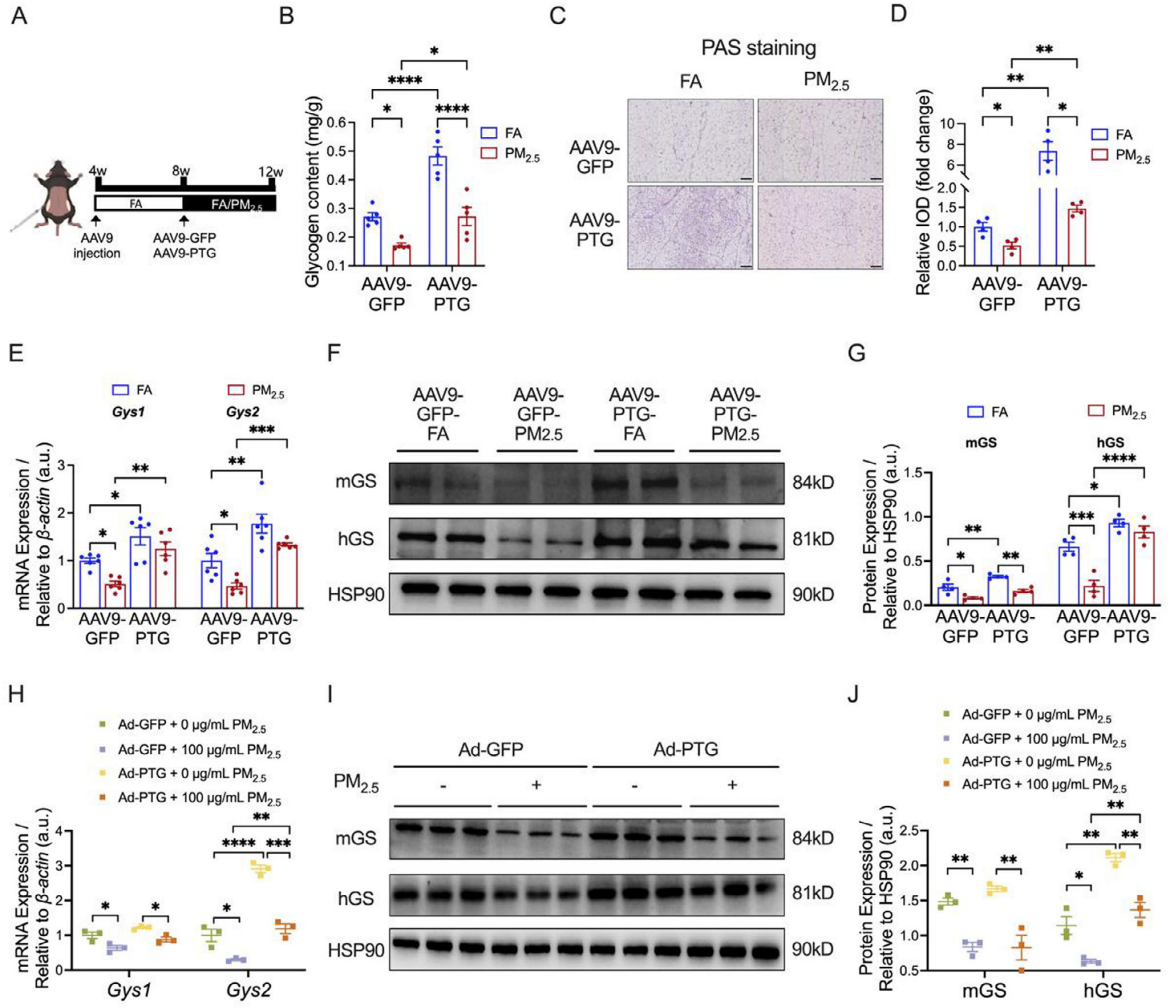
Effects of PTG overexpression on PM_2.5_‐induced glycogen metabolism dysregulation in iWAT. (A) Schematic representation of AAV9‐PTG mice by AAV9 injection. (B) Glycogen content in iWAT (*n* = 5 per group). (C,D) Representative images (C) and relative IOD (D) of PAS staining in iWAT sections (*n* = 4 per group); Scale bar = 50 µm. (E) mRNA expression of rate‐limiting enzymes of glycogen synthesis in iWAT (*n* = 6 per group). (F,G) Representative bands (F) and quantitative analysis (G) of rate‐limiting enzymes of glycogen synthesis in iWAT (*n* = 4 per group). (H) mRNA expression of rate‐limiting enzymes of glycogen synthesis in 3T3‐L1 adipocytes (*n* = 3 per group). (I,J) Representative bands (I) and quantitative analysis (J) of rate‐limiting enzymes of glycogen synthesis in 3T3‐L1 adipocytes (*n* = 3 per group). Data were presented as mean ± SEM, analyzed by two‐way ANOVA with Tukey's multiple comparisons test. ^*^ *p* < 0.05, ^**^ *p* < 0.01, ^***^ *p* < 0.001, ^****^ *p* < 0.0001.

We established a specific PTG overexpression mouse model (AAV9‐PTG) through orthotopic injection of an AAV9, followed by PM_2.5_ exposure (Figure 4A). We verified that the mRNA levels of *Ppp1r3c* in iWAT of AAV9‐PTG mice increased approximately 106‐fold (Figure S2A), along with a significant upregulation of PTG protein levels (Figure S2B,C), confirming successful model construction. Subsequently, we investigated the regulatory impact of PTG overexpression on glycogen metabolism in iWAT of PM_2.5_‐exposed mice. The findings indicated that PTG overexpression notably elevated the glycogen content in iWAT of mice and effectively mitigated the reduction in glycogen content induced by PM_2.5_ exposure (Figure 4B). This phenomenon was further corroborated by PAS staining (Figure 4C,D). PTG overexpression significantly upregulated the mRNA expression levels of glycogen synthase isoforms (*Gys1*, *Gys2*), and effectively counteracted PM_2.5_‐induced reduction of these genes in iWAT of mice (Figure 4E). Furthermore, it also significantly increased the protein expression levels of mGS and hGS in the iWAT of mice (Figure 4F,G). Additionally, it effectively reversed the downregulation of hGS protein expression caused by PM_2.5_ exposure (Figure 4F,G).

In 3T3‐L1 adipocytes, we established a PTG overexpression (Ad‐PTG) cell model through adenovirus transfection, and then exposed them to PM_2.5_. We verified that in Ad‐PTG cells, the mRNA levels of *Ppp1r3c* increased approximately 10‐fold (Figure S3A), accompanied by a marked elevation in PTG protein levels (Figure S3B,C). These results demonstrated the successful establishment of the PTG overexpression cell model. PTG overexpression significantly increased the mRNA and protein levels of hGS and reversed PM_2.5_‐induced hGS downregulation in 3T3‐L1 adipocytes (Figure 4H–J).

Collectively, these findings suggested that PTG overexpression significantly enhanced the glycogen content in iWAT inhibited by PM_2.5_ exposure via modulating the expression of glycogen synthesis enzymes (particularly hGS).

### 2.5 PTG Overexpression Attenuated PM_2.5_‐Induced Systemic Metabolic Dysfunctions and Impaired iWAT Browning

**FIGURE 5:**
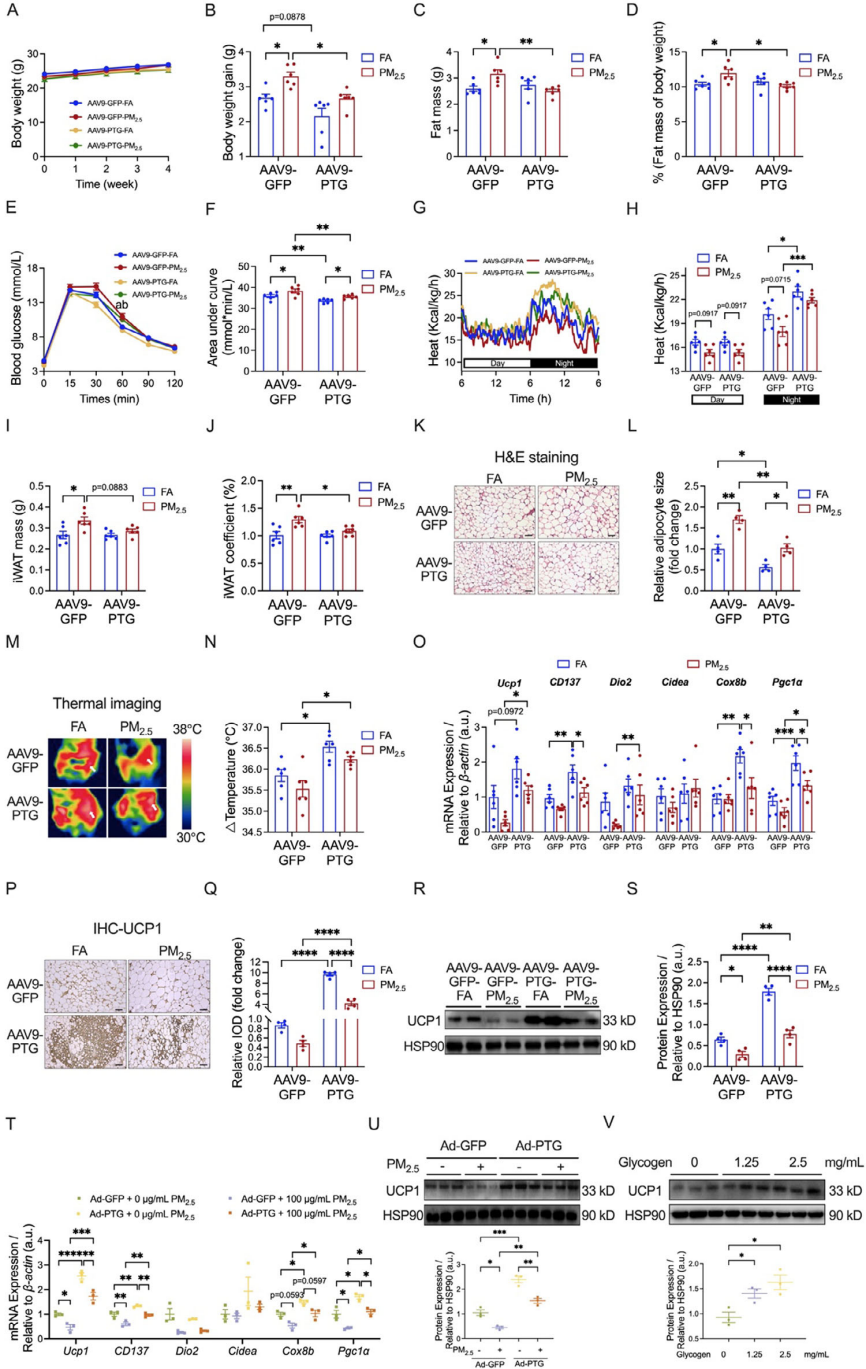
Effects of PTG overexpression on PM_2.5_‐induced systemic metabolic dysfunctions and impaired iWAT browning. (A,B) Body weight (A) and body weight gain (B) of mice (*n* = 6 per group). (C,D) Fat mass (C) and the ratio of fat mass to body weight (D) of mice (*n* = 6 per group). (E,F) GTT (E) and AUC of GTT (F) of mice (*n* = 6 per group). (G,H) Heat production of mice (*n* = 6 per group). Line graph (G) indicates variation patterns of heat production of mice over a 24 h period, grouped column graph (H) indicates the mean value at the time points indicated. (I,J) iWAT mass (I) and iWAT coefficient (J) of mice (*n* = 6 per group). (K,L) Representative images (K) and quantitative analysis (L) of H&E staining in iWAT sections (*n* = 4 per group); Scale bar = 50 µm. (M,N) Representative thermographic camera images (M) as shown by FLIR image and quantification of temperature changes (N) in iWAT. The white arrow indicates the inguinal area of the abdomen (*n* = 6 per group). (O) mRNA expression of thermogenic genes in iWAT (*n* = 6 per group). (P,Q) Representative images (P) and relative IOD (Q) of immunohistochemistry in iWAT sections stained with UCP1 antibody (*n* = 4 per group); Scale bar = 50 µm. (R,S) Representative bands (R) and quantitative analysis (S) of UCP1 in iWAT (*n* = 4 per group). (T) mRNA expression of thermogenic genes in 3T3‐L1 adipocytes (*n* = 3 per group). (U) Representative bands (top) and quantitative analysis (bottom) of UCP1 in 3T3‐L1 adipocytes (*n* = 3 per group). (V) Representative bands (top) and quantitative analysis (bottom) of UCP1 in 3T3‐L1 adipocytes (*n* = 3 per group). Data were presented as mean ± SEM, analyzed by two‐way ANOVA with Tukey's multiple comparisons test (A–U) or one‐way ANOVA with Tukey's test (V). ^a^ *p* < 0.05 AAV9‐GFP‐FA vs AAV9‐GFP‐PM_2.5_, ^b^ *p* < 0.05 AAV9‐PTG‐FA vs AAV9‐PTG‐PM_2.5_. ^*^ *p* < 0.05, ^**^ *p* < 0.01, ^***^ *p* < 0.001, ^****^ *p* < 0.0001.

We investigated the impact of PTG overexpression on systemic metabolism in PM_2.5_‐exposed mice. Although the trajectories of body weight did not differ among the groups (Figure 5A), PTG overexpression notably suppressed body weight gain in the mice and effectively mitigated the PM_2.5_‐induced increase in body weight gain (Figure 5B). The increases in fat mass (Figure 5C) and its ratio to body weight (Figure 5D) induced by PM_2.5_ exposure were significantly attenuated in PTG‐overexpressing mice. Additionally, PTG overexpression not only significantly enhanced glucose tolerance but also reversed the impaired glucose tolerance induced by PM_2.5_ exposure in mice (Figure 5E,F). Further analysis of energy metabolism revealed that PTG overexpression exerted no significant effect on daytime total heat production, whereas it significantly increased the total heat production at the peak of nighttime activity and alleviated the decrease in heat production caused by PM_2.5_ exposure (Figure 5G,H). These findings suggested that PTG overexpression could significantly ameliorate systemic metabolic disorders induced by PM_2.5_ exposure in mice, including fat metabolism, glucose metabolism, and energy metabolism.

We further assessed the impact of PTG overexpression on the thermogenic function of iWAT in PM_2.5_‐exposed mice. The increase in iWAT mass (Figure 5I) and organ coefficient (Figure 5J) induced by PM_2.5_ exposure was effectively mitigated by PTG overexpression. H&E staining further confirmed that PTG overexpression significantly reduced the size of adipocytes in iWAT and effectively reversed the cell hypertrophy induced by PM_2.5_ exposure (Figure 5K,L). The results of infrared thermal imaging revealed that PTG overexpression significantly increased the local temperature of iWAT in both FA‐ and PM_2.5_‐exposed mice, compared with their respective controls (Figure 5M,N). qRT‐PCR results showed that PTG overexpression significantly upregulated the mRNA expression levels of *Ucp1*, *CD137*, *Cox8b*, and *Pgc1α* (Figure 5O). The mRNA expression levels of *Ucp1*, *Dio2*, and *Pgc1α* in the AAV9‐PTG‐PM_2.5_ group were significantly higher than those in the AAV9‐GFP‐PM_2.5_ group (Figure 5O). Immunohistochemical (Figure 5P,Q) and western blotting (Figure 5R,S) results further confirmed that PTG overexpression significantly upregulated UCP1 protein expression levels and effectively reversed the decrease of UCP1 expression induced by PM_2.5_ exposure. These results indicated that PTG overexpression significantly improved PM_2.5_‐impaired thermogenesis of iWAT in mice.

Subsequently, we conducted validation at the cellular level. PTG overexpression significantly increased the mRNA expression levels of *Ucp1*, *CD137*, *Cox8b*, and *Pgc1α* in 3T3‐L1 adipocytes (Figure 5T). Moreover, it effectively counteracted the PM_2.5_‐inhibited expression of these genes, though the levels of *Dio2* and *Cidea* remained unchanged (Figure 5T). Western blotting analysis further confirmed that PTG overexpression notably increased the UCP1 protein expression and effectively reversed the reduction of UCP1 protein level in 3T3‐L1 adipocytes triggered by PM_2.5_ exposure (Figure 5U). These findings suggested that PTG overexpression could significantly alleviate the inhibitory effect of PM_2.5_ exposure on the thermogenic function of 3T3‐L1 adipocytes.

To further verify the role of glycogen in the browning process of iWAT, 3T3‐L1 adipocytes were treated with different concentrations of glycogen. As shown in Figure 5V, as the glycogen concentration increased, the expression level of the UCP1 protein in 3T3‐L1 adipocytes increased significantly. These findings suggested that increased glycogen content could significantly promote the browning process of 3T3‐L1 adipocytes, and further confirmed the molecular mechanism by which PTG plays an important role in the browning of iWAT by regulating glycogen metabolism.

### 2.6 PTG Overexpression Mitigated PM_2.5_‐Induced Mitochondrial Dysfunction in iWAT

**FIGURE 6:**
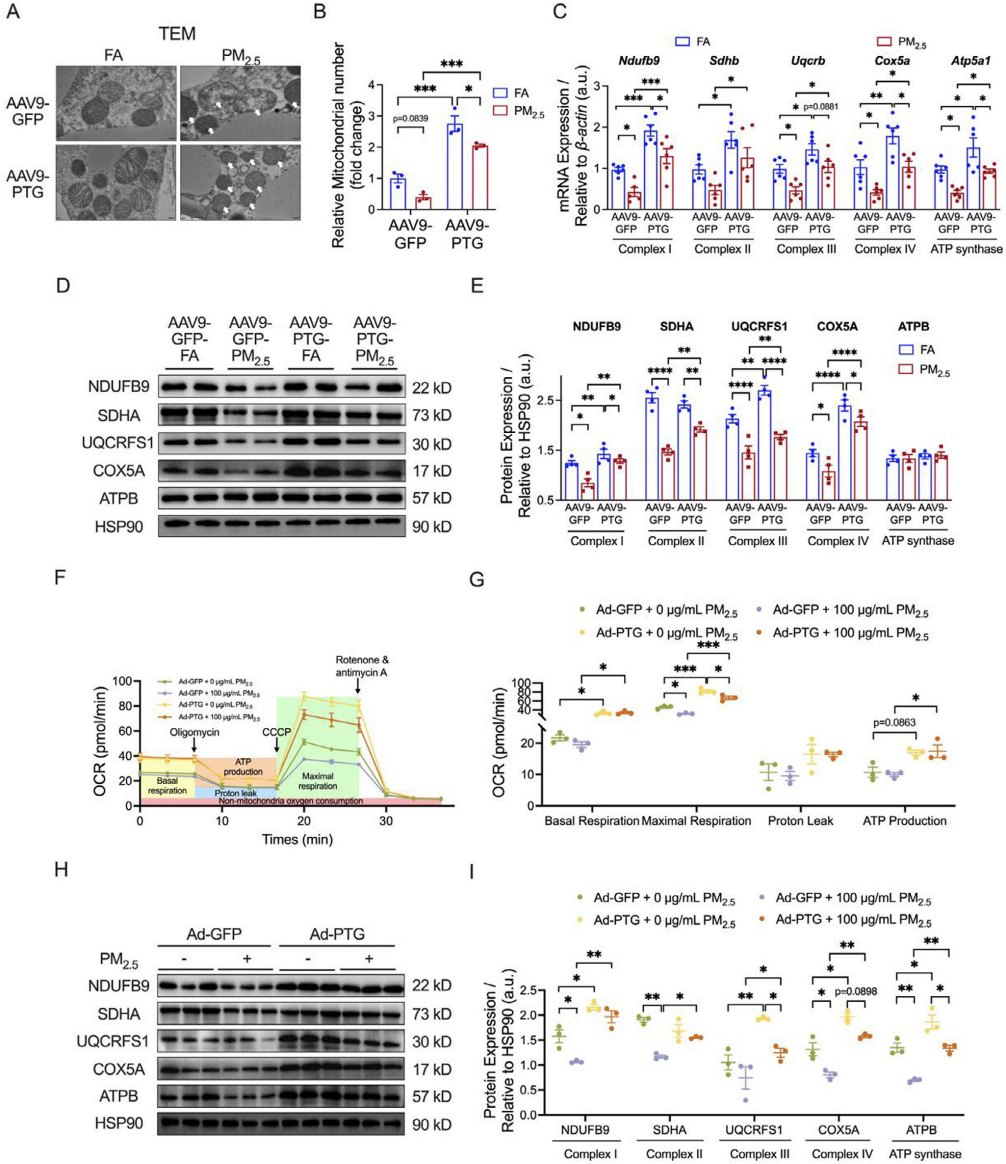
Effects of PTG overexpression on PM_2.5_‐induced mitochondrial dysfunction in iWAT. (A,B) Representative transmission electron microscopy images (A) and quantification of relative mitochondrial number (B) in iWAT sections (*n* = 3 per group); Scale bar = 200 nm. (C) mRNA expression of Complex I‐IV and ATP synthase subunits‐related genes in iWAT (*n* = 6 per group). (D,E) Representative bands (D) and quantitative analysis (E) of NDUFB9, SDHA, UQCRFS1, COX5A, and ATPB in iWAT (*n* = 4 per group). (F,G) OCR (F) and indicators of mitochondrial respiratory function (G) of 3T3‐L1 adipocytes (*n* = 3 per group). (H,I) Representative bands (H) and quantitative analysis (I) of NDUFB9, SDHA, UQCRFS1, COX5A, and ATPB in 3T3‐L1 adipocytes (*n* = 3 per group). Data were presented as mean ± SEM, analyzed by two‐way ANOVA with Tukey's multiple comparisons test. ^*^ *p* < 0.05, ^**^ *p* < 0.01, ^***^ *p* < 0.001, ^****^ *p* < 0.0001.

Mitochondria, serving as the core site of cellular energy metabolism, play a pivotal role in the browning process of iWAT [36, 37]. We further investigated the impact of PTG overexpression on mitochondrial function in iWAT of PM_2.5_‐exposed mice. Transmission electron microscopy revealed that PM_2.5_ exposure led to disordered mitochondrial cristae structure in iWAT, whereas PTG overexpression significantly improved the integrity of mitochondrial cristae structure (Figure 6A). It also increased the number of mitochondria in iWAT and effectively alleviated the reduction in mitochondrial number induced by PM_2.5_ exposure (Figure 6A,B). Subsequently, we analyzed the expression levels of mitochondrial oxidative phosphorylation (OXPHOS) components, including complexes I‐IV and ATP synthase (complex V). PTG overexpression significantly upregulated the mRNA expression levels of OXPHOS‐related genes, including NADH dehydrogenase iron‐sulfur protein 9 (*Ndufb9*), succinate dehydrogenase complex subunit B (*Sdhb*), ubiquinol‐cytochrome c reductase R subunit B (*Uqcrb*), cytochrome c oxidase subunit 5A (*Cox5a*), and ATP synthase subunit 5A1 (*Atp5a1*) (Figure 6C). Notably, PTG overexpression significantly reversed the PM_2.5_‐induced downregulation of *Ndufb9*, *Uqcrb*, *Cox5a*, and *Atp5a1* mRNA expression (Figure 6C). Western blotting further confirmed that the protein expression levels of NDUFB9, UQCRFS1, and COX5A were significantly higher in the AAV9‐PTG‐FA group than in the AAV9‐GFP‐FA group (Figure 6D,E). Meanwhile, the protein expression levels of NDUFB9, SDHA, UQCFS1, and COX5A were downregulated by PM_2.5_ exposure, which were significantly increased after PTG overexpression, while the expression level of ATPB was not significantly changed (Figure 6D,E).

Then, we validated the results at the cellular level. The O2K examination showed that PTG overexpression significantly increased basal respiration, maximal respiration, and ATP production of 3T3‐L1 adipocytes under either 0 µg/mL or 100 µg/mL PM_2.5_ treatment conditions (Figure 6F,G). Notably, PTG overexpression significantly alleviated the decrease in maximal respiration caused by PM_2.5_ exposure (Figure 6F,G). However, no significant differences in the proton leak levels were found among the groups (Figure 6F,G). Moreover, PTG overexpression significantly upregulated the protein levels of NDUFB9, UQCRFS1, COX5A, and ATPB, and effectively ameliorated PM_2.5_‐induced decreases in NDUFB9, SDHA, COX5A, and ATPB protein levels (Figure 6H‐I).

In conclusion, PTG overexpression played a crucial role in maintaining the browning of iWAT following PM_2.5_ exposure by enhancing mitochondrial morphology, number, and function.

### 2.7 VEGFB Mediated the Protective Effects of PTG Overexpression Against PM_2.5_‐Impaired iWAT Browning

**FIGURE 7:**
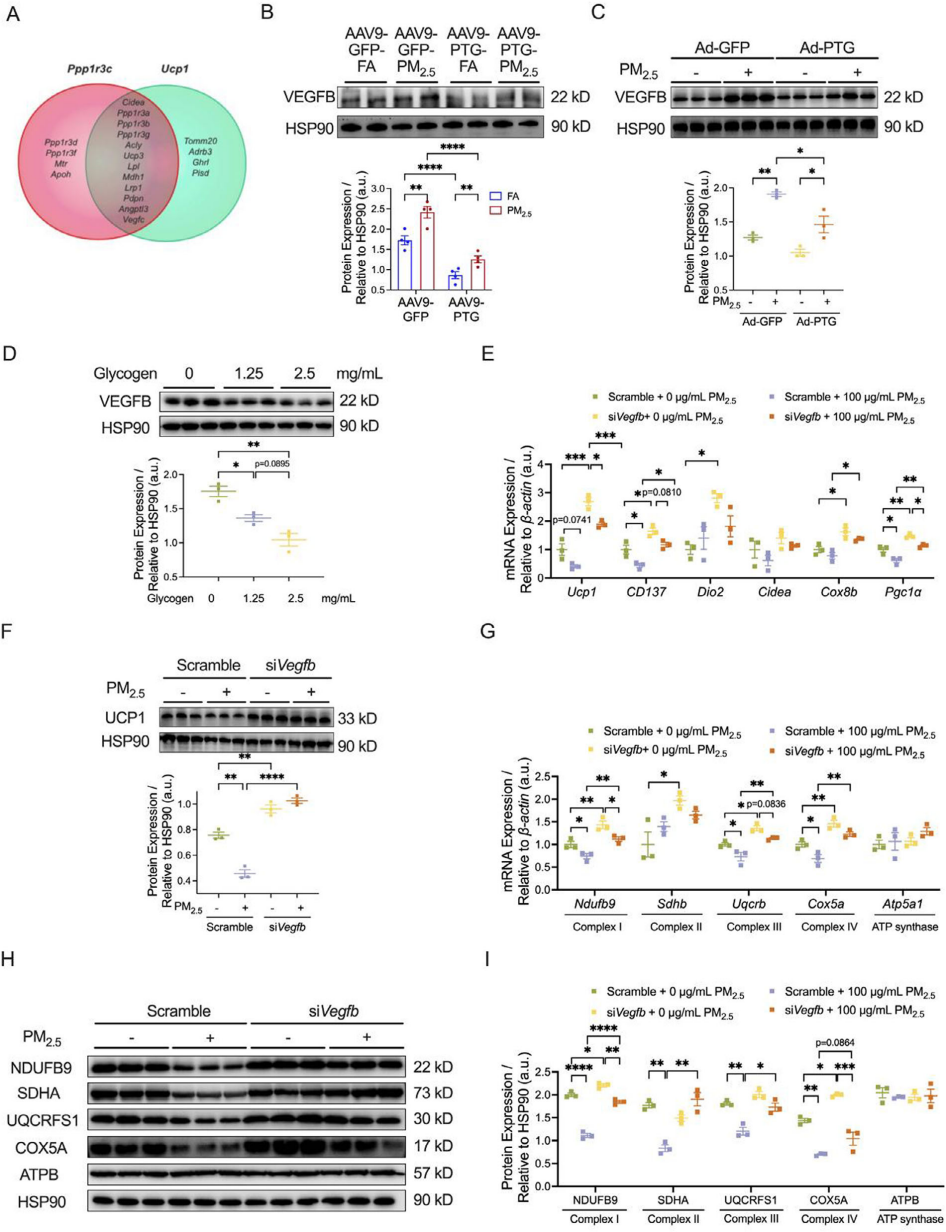
VEGFB mediated the protective effects of PTG overexpression against PM_2.5_‐impaired iWAT browning. (A) Target analysis of *Ppp1r3c* and *Ucp1* by GeneMANIA. (B) Representative bands (top) and quantitative analysis (bottom) of VEGFB in iWAT (*n* = 4 per group). (C) Representative bands (top) and quantitative analysis (bottom) of VEGFB in 3T3‐L1 adipocytes (*n* = 3 per group). (D) Representative bands (top) and quantitative analysis (bottom) of VEGFB in 3T3‐L1 adipocytes (*n* = 3 per group). (E) mRNA expression of thermogenic genes in 3T3‐L1 adipocytes (*n* = 3 per group). (F) Representative bands (top) and quantitative analysis (bottom) of UCP1 in 3T3‐L1 adipocytes (*n* = 3 per group). (G) mRNA expression of Complex I‐IV and ATP synthase subunits‐related genes in 3T3‐L1 adipocytes (*n* = 3 per group). (H,I) Representative bands (H) and quantitative analysis (I) of NDUFB9, SDHA, UQCRFS1, COX5A, and ATPB in 3T3‐L1 adipocytes (*n* = 3 per group). Data were presented as mean ± SEM, analyzed by two‐way ANOVA with Tukey's multiple comparisons test (B,C, E–I) or one‐way ANOVA with Tukey's test (D). ^*^ *p* < 0.05, ^**^ *p* < 0.01, ^***^ *p* < 0.001, ^****^ *p* < 0.0001.

To elucidate the mechanism of glycogen metabolism in the inhibition of iWAT browning by PM_2.5_ exposure, gene interaction network analysis of core molecules *Ppp1r3c* and *Ucp1* was performed using the GeneMANIA database. From the resulting network, a shortlist of candidate molecules, including vascular endothelial growth factor (VEGF) was identified as the potential molecular targets (Figure 7A). VEGFB (a member of the VEGF family), but not other molecules, was validated to be the key regulator of glycogen metabolism (Figure S4A,B). It was further confirmed by the findings that PTG overexpression significantly reduced VEGFB protein levels in iWAT (Figure S5A) and 3T3‐L1 adipocytes (Figure S5B), whereas PTG knockdown increased VEGFB expression in 3T3‐L1 adipocytes (Figure S5C). Notably, PM_2.5_ exposure upregulated VEGFB protein levels in both iWAT (Figure 7B) and 3T3‐L1 adipocytes (Figure 7C), an effect counteracted by PTG overexpression (Figure 7B,C). Furthermore, elevated glycogen concentrations suppressed VEGFB protein expression in 3T3‐L1 adipocytes (Figure 7D), whereas VEGFB knockdown did not alter PTG expression (Figure S5D), clearly indicating the regulation of PTG on VEGFB expression via glycogen.

To clarify the role of VEGFB in the inhibition of iWAT browning in response to PM_2.5_ exposure, we established a VEGFB knockdown cell model through siRNA transfection (Figure S6A–C). VEGFB knockdown significantly upregulated the mRNA levels of *Ucp1*, *CD137*, *Dio2*, *Cox8b*, and *Pgc1α* (Figure 7E). Moreover, it effectively counteracted PM_2.5_‐induced downregulation of *Ucp1*, *CD137*, and *Pgc1α* mRNA levels (Figure 7E). Western blotting analysis further revealed that VEGFB knockdown itself led to a significant increase in the protein level of UCP1 and significantly reversed the inhibition of PM_2.5_ exposure on UCP1 expression (Figure 7F). Moreover, VEGFB knockdown significantly elevated the mRNA levels of molecules for OXPHOS, including *Ndufb9*, *Sdhb*, *Uqcrb*, and *Cox5a*, and effectively alleviated the PM_2.5_‐induced decrease of *Ndufb9*, *Uqcrb*, and *Cox5a* (Figure 7G). At the protein level, VEGFB knockdown only increased the protein expression of NDUFB9 and COX5A, whereas it significantly ameliorated the PM_2.5_‐induced downregulation of NDUFB9, SDHA, UQCRFS1, and COX5A protein expression (Figure 7H,I). However, expression levels of either *Atp5a1* mRNA or ATPB protein remained unchanged among the groups (Figure 7G–I). In summary, VEGFB knockdown could effectively alleviate the inhibitory effect of PM_2.5_ exposure on the browning of 3T3‐L1 adipocytes by enhancing thermogenic function and improving mitochondrial oxidative phosphorylation.

### 2.8 ADRB3 Acted as an Upstream Regulator of PTG in PM_2.5_‐Inhibited iWAT Browning

**FIGURE 8:**
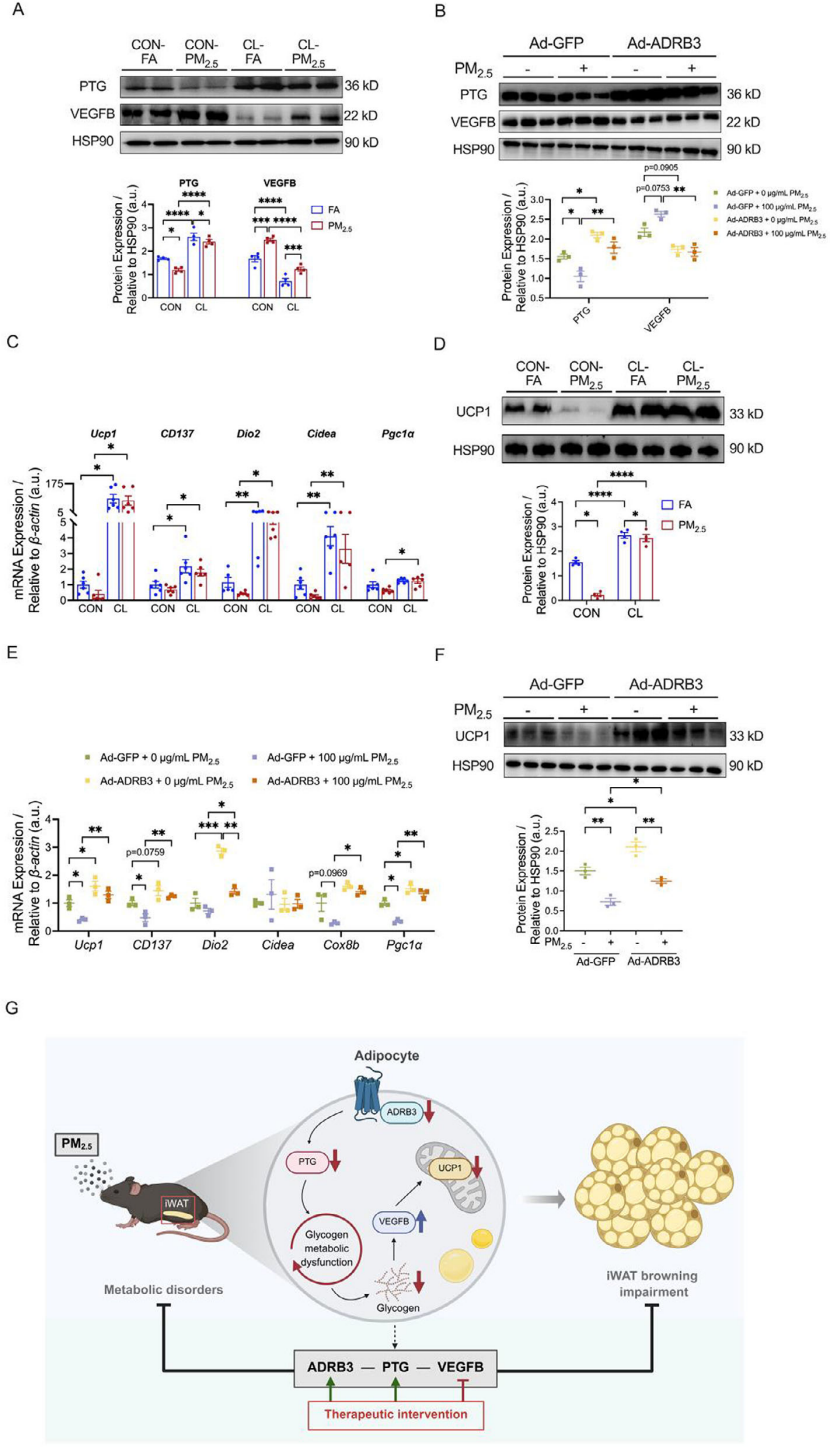
ADRB3 acted as an upstream regulator of PTG in PM_2.5_‐inhibited iWAT browning. (A) Representative bands (top) and quantitative analysis (bottom) of PTG and VEGFB in iWAT (*n* = 4 per group). (B) Representative bands (top) and quantitative analysis (bottom) of PTG and VEGFB in 3T3‐L1 adipocytes (*n* = 3 per group). (C) mRNA expression of thermogenic genes in iWAT (*n* = 6 per group). (D) Representative bands (top) and quantitative analysis (bottom) of UCP1 in iWAT (*n* = 4 per group). (E) mRNA expression of thermogenic genes in 3T3‐L1 adipocytes (*n* = 3 per group). (F) Representative bands (top) and quantitative analysis (bottom) of UCP1 in 3T3‐L1 adipocytes (*n* = 3 per group). (G) Schematic illustration of proposed mechanisms. This schematic illustrates the pathway linking PM_2.5_ exposure to impaired iWAT browning through PTG‐dependent glycogen metabolism dysregulation. Data were presented as mean ± SEM, analyzed by two‐way ANOVA with Tukey's multiple comparisons test. ^*^ *p* < 0.05, ^**^ *p* < 0.01, ^***^ *p* < 0.001, ^****^ *p* < 0.0001.

Our previous findings indicated that ADRB3 signaling represents a crucial molecular mechanism underlying the impaired iWAT browning by PM_2.5_ exposure [20]. We next analyzed whether ADRB3 is involved in PM_2.5_‐inhibited iWAT browning via regulation of PTG and VEGFB. Using a mouse model with CL316243‐upregulated ADRB3 signaling (Figure S7A,B) and a 3T3‐L1 cell model with ADRB3 overexpression (Figure S8A–C), we demonstrated that both activation (Figure S9A) and overexpression (Figure S9B) of ADRB3 significantly upregulated the protein expression level of PTG and glycogen content in 3T3‐L1 adipocytes (Figure S10), whereas it significantly downregulated that of VEGFB (Figure S9A,B). Further studies showed that PM_2.5_‐downregulated PTG protein levels were significantly reversed in both ADRB3 agonist‐treated mouse model (Figure 8A) and ADRB3‐overexpressing cellular model (Figure 8B). Similarly, PM_2.5_ exposure significantly upregulated protein expression of VEGFB, which was reversed by ADRB3 activation (Figure 8A) and overexpression (Figure 8B) as well. However, PTG overexpression did not alter the protein level of ADRB3 in either iWAT (Figure S9C) or adipocytes (Figure S9D). Collectively, these results suggested that ADRB3 might serve as an upstream regulator of PTG, positively modulating PTG expression, followed by inhibition of VEGFB expression.

Subsequently, we comprehensively assessed the impact of enhanced ADRB3 activity on iWAT browning in mice exposed to PM_2.5_. Under both FA and PM_2.5_ exposure conditions, ADRB3 activation significantly upregulated the mRNA expression levels of *Ucp1*, *CD137*, *Dio2*, and *Cidea* in iWAT of mice (Figure 8C). Western blotting further corroborated that increased ADRB3 activity not only significantly elevated the protein level of UCP1 in iWAT of mice but also effectively reversed the downregulation of UCP1 protein expression induced by PM_2.5_ exposure (Figure 8D). In 3T3‐L1 adipocytes, ADRB3 overexpression significantly upregulated the mRNA levels of *Ucp1*, *CD137*, *Dio2*, and *Pgc1α* (Figure 8E). Moreover, it effectively reversed the decrease of *Ucp1*, *CD137*, *Cox8b*, and *Pgc1α* gene expression caused by PM_2.5_ exposure (Figure 8E). Western blotting further confirmed that ADRB3 overexpression significantly increased the protein level of UCP1 in 3T3‐L1 adipocytes and mitigated its downregulation induced by PM_2.5_ exposure (Figure 8F). These findings suggested that ADRB3 activation and overexpression could ameliorate the dysfunction of iWAT browning induced by PM_2.5_ exposure via the PTG‐VEGFB signaling pathway.

## 3 Discussion

This study provides mechanistic insights into how PM_2.5_ exposure disrupts glycogen metabolism to suppress iWAT browning, offering new perspectives for addressing PM_2.5_‐associated metabolic disorders. Building upon our previous findings demonstrating PM_2.5_‐induced inhibition of iWAT browning [20] and metabolic dysfunction, we now present the following key discoveries: (1) PM_2.5_ exposure significantly impaired glycogen metabolism in iWAT. (2) PTG overexpression not only rescued PM_2.5_‐induced glycogen metabolic dysregulation in iWAT, but also mitigated thermogenic impairment, mitochondrial dysfunction, and compromised browning capacity in iWAT, along with systemic metabolic dysfunctions. (3) ADRB3‐PTG‐VEGFB signaling axis was the mechanistic framework in PM_2.5_‐induced metabolic defects and adipose browning suppression (Figure 8G). These findings represent the first evidence that PM_2.5_ disrupted adipose browning through glycogen metabolic dysregulation. Importantly, our work unveils a novel “pollution‐profiling‐protection” paradigm by highlighting PTG‐mediated glycogen metabolism as the core for counteracting the metabolic consequences of air pollution exposure.

Our study revealed for the first time that PM_2.5_ exposure substantially impaired glycogen metabolism in iWAT. Generally, adipose tissue typically contains substantially lower glycogen levels than liver or skeletal muscle [38, 39], rendering adipose glycogen largely neglected in metabolic disorders. However, adipose glycogen exhibited dynamic fluctuations during metabolic challenges, showing marked depletion after 48‐h fasting and rapid replenishment upon refeeding [25], while acute leptin deficiency triggers transient glycogen accumulation in lipid‐depleted adipocytes [40], collectively indicating the potential role of adipose glycogen in metabolic regulation. Clinically relevant observations showed that enhanced adipose glycogen synthesis correlated with improved body weight control and insulin sensitivity across both sexes [28]. Although PM_2.5_‐induced hepatic glycogen depletion and its metabolic consequences (insulin resistance and diabetes‐related metabolic complications) were well‐documented [23, 41, 42], the pollutant's effects on iWAT glycogen remained unexplored until now. Our discovery of PM_2.5_‐mediated glycogen reduction in iWAT, coupled with the strong association of glycogen content with UCP1 expression, suggests that glycogen may be a potential biomarker for PM_2.5_‐driven metabolic disorder. Further intervention with glycogen restoration rescued PM_2.5_‐impaired thermogenesis, establishing a novel mechanistic link between air pollution and metabolic dysfunction through adipose tissue glycogen metabolism.

Another novelty of the study was that PTG overexpression not only rescued PM_2.5_‐induced glycogen metabolism disorders but also exerted a broad‐spectrum metabolic protection against PM_2.5_ exposure. As a critical glycogen‐binding subunit of PP1 [27, 43, 44], PTG enhanced glycogen synthesis in iWAT through increasing expression of glycogen synthase (hGS), effectively counteracting PM_2.5_‐induced glycogen depletion. This data mirrored observations in human adipocytes where PTG overexpression elevated glycogen content and modulated adipocyte metabolism [45]. The clinical relevance of these findings was underscored by the negative correlation between iWAT PTG levels and key metabolic parameters (waist circumference, body weight, and insulin resistance index) in both obese and non‐obese individuals [28]. Consistently, our study revealed that iWAT‐specific PTG overexpression prevented PM_2.5_‐induced body weight gain, adipose tissue accumulation, and glucose intolerance.

We further established that PTG overexpression completely blocked PM_2.5_‐induced suppression of iWAT browning. This finding built upon previous work showing reduced UCP1 expression in iWAT‐specific PTG knockout mice [28], directly linking PTG‐mediated glycogen metabolism to thermogenic function in iWAT. Our results significantly extended the understanding of the setting of PM_2.5_ exposure by demonstrating the following evidence. PM_2.5_‐inhibited systemic heat production and local temperature of iWAT in mice were completely blocked by iWAT‐specific PTG overexpression. Additionally, the thermogenic and mitochondrial protective effects against PM_2.5_ exposure occurred both in vivo (iWAT) and in vitro (adipocytes) in the presence of PTG overexpression. Most importantly, exogenous glycogen supplementation alone upregulated UCP1 expression in adipocytes, directly establishing PTG‐mediated glycogen metabolism as a regulator of iWAT browning.

Through comprehensive screening, we identified VEGFB as a novel downstream target of PTG that mediated its protective effects against PM_2.5_‐induced suppression of iWAT browning. While primarily recognized for its role in vascular development [46], VEGFB has emerged as an important regulator of energy metabolism [47, 48]. Elevated serum VEGFB levels correlated with obesity [49] and type 2 diabetes complications [50], whereas VEGFB knockdown promoted the browning of iWAT in mice [51], indicating important clinical and mechanistic implications. Notably, in obese adipose‐specific GSK3β (a negative regulator of glycogen synthase activity) knockout mice, elevated VEGFB protein levels in eWAT were observed [52], suggesting a potential regulatory relationship between glycogen metabolism and VEGFB expression. Our findings demonstrated that PTG overexpression effectively counteracted PM_2.5_‐induced VEGFB upregulation in both mouse iWAT and 3T3‐L1 adipocytes. In addition, exogenous glycogen supplementation alone was sufficient to reduce VEGFB expression, whereas VEGFB silencing reversed PM_2.5_‐induced browning inhibition in 3T3‐L1 adipocytes. These findings indicated that VEGFB may be a potential biomarker in response to environmental insult (PM_2.5_) and the target on which PTG‐mediated glycogen protects against PM_2.5_‐suppressed iWAT browning. The biological effects of VEGFB are mediated through its binding to VEGFR1 [53], which is predominantly expressed in endothelial cells [54, 55]. Importantly, endothelial‐specific VEGFR1 knockdown has been shown to improve metabolic function by inducing iWAT browning [51, 56]. These findings collectively support our hypothesis that PTG may alleviate PM_2.5_‐induced iWAT browning impairment through modulation of the VEGFB‐VEGFR1 signaling axis, although further experimental validation is warranted.

Our study elucidated a critical signaling hierarchy in which ADRB3 acted as the primary upstream regulator of PTG in PM_2.5_‐mediated browning inhibition. As the dominant receptor governing adaptive thermogenesis in brown and beige adipocytes [29, 57, 58], ADRB3's connection to glycogen metabolism was first suggested by Markan et al., who demonstrated β‐adrenergic enhancement of glycogen turnover in PTG transgenic models [31]. Based on the finding that CL316243 (ADRB3‐specific agonist) treatment upregulated key glycogen metabolism genes (*Gys1* and *Ppp1r3c*, which encode mGS and PTG, respectively) in mouse iWAT [28], we further provided strong evidence showing PTG protein upregulation in both ADRB3‐activated mice and ADRB3‐overexpressing 3T3‐L1 adipocytes, establishing ADRB3‐PTG regulatory hierarchy.

It has been reported that isoproterenol significantly increased VEGF expression by activating ADRB2 in non‐small cell lung cancer cells [59], suggesting a close link between the ADRB signaling pathway and VEGF expression. Our findings revealed that VEGFB protein expression was significantly downregulated in response to both ADRB3 activation and overexpression. These results suggested that the ADRB3 signaling pathway may participate in the metabolic regulation process of adipose tissue by regulating VEGFB expression. Building upon the well‐characterized membrane localization of ADRB3 and its canonical role in UCP1‐mediated thermogenesis [60, 61], we extended to confirm that ADRB3 activation and overexpression, PTG overexpression, and VEGFB silencing all effectively reversed the inhibition of iWAT browning (including UCP1 expression) induced by PM_2.5_ exposure. Taken together, we established a definitive signaling cascade of the ADRB3‐PTG‐VEGFB axis as the mechanistic framework, in which PM_2.5_ exposure disrupted iWAT browning suppression and metabolic defects.

As an extension of our previous study, it is notable that the current study was conducted exclusively in male mice, thereby eliminating any confounding effects from cyclical hormonal fluctuations. However, given the well‐documented sexual dimorphism in adipose tissue distribution, thermogenic capacity, and immune responses, the generalizability of our findings to females requires direct investigation. Future studies employing both male and female models are warranted to elucidate the potential influence of sex hormones and chromosomal differences on PM_2.5_’s disruption of glycogen metabolism and adipose browning.

In summary, the present study provided the first evidence to show that PM_2.5_ impaired iWAT browning via PTG‐mediated glycogen metabolism disruption, which was initiated by ADRB3 inhibition and subsequently triggered VEGFB upregulation. While this study delineated the mechanism in a pre‐clinical model, our findings offered valuable insights into understanding the metabolic risks posed by PM_2.5_ exposure in humans. It posited that impairment in adipose tissue glycogen metabolism and its consequent suppression of browning capacity could be a previously overlooked pathway contributing to environment‐associated metabolic syndromes, such as obesity and insulin resistance, in human populations. The established ADRB3‐PTG‐VEGFB mechanistic framework represents a paradigm shift in environmental medicine, moving beyond observational associations to a translational framework for biomarker development and therapeutic intervention.

## 4 Methods

### 4.1 In Vivo Studies‐Animal and PM_2.5_ Exposure

Male C57BL/6 mice were purchased from Charles River Laboratory (Charles River, Beijing, China) and were kept in standard cages at a regulated temperature of 22 ± 2°C, following a 12‐h light and 12‐h dark rhythm. All mice received a regular chow diet ad libitum. The Institutional Animal Care and Use Committee at Zhejiang Chinese Medical University (ZCMU) granted approval for all animal procedures associated with this research (IACUC‐202409‐23). All animal experiments followed the Animal Research: Reporting of In Vivo Experiments (ARRIVE) guidelines.

To investigate the effects of PM_2.5_ exposure on iWAT browning and glycogen metabolism in mice, 8‐week‐old male mice were randomly divided into the FA control group and the concentrated PM_2.5_ exposure group. The Zhejiang Whole‐body Exposure System (ZJ‐WES) located on the campus of ZCMU was employed to establish exposure models for a duration of 4 consecutive weeks, with a daily exposure time of 12 h per day. The principle of the ZJ‐WES was described previously [62, 63], and the PM_2.5_ exposure condition was set based on previous studies [32].

To explore the role of glycogen metabolism in the inhibition of iWAT browning by PM_2.5_ exposure, 8‐week‐old male control (AAV9‐GFP) mice and PTG overexpression (AAV9‐PTG) mice were randomly divided into four groups: AAV9‐GFP‐FA; AAV9‐GFP‐PM_2.5_; AAV9‐PTG‐FA; AAV9‐PTG‐PM_2.5_. The ZJ‐WES was used for exposure modeling for 4 consecutive weeks, and the exposure time was 12 h per day.

To explore the role of ADRB3 in PTG‐mediated inhibition of iWAT browning by PM_2.5_ exposure, 8‐week‐old male mice were randomly divided into four groups: CON‐FA; CON‐PM_2.5_; CL‐FA; CL‐PM_2.5_. The ZJ‐WES was used to establish the exposure model for 4 consecutive weeks, and the exposure time was 12 h per day. Based on our previous studies [20], after 3‐week PM_2.5_ exposure, CL‐FA and CL‐PM_2.5_ groups were intraperitoneally injected with CL316243 at a dose of 1 mg/kg daily for 7 consecutive days. CON‐FA and CON‐PM_2.5_ groups received daily intraperitoneal injection of an equal volume of normal saline as a control.

### 4.2 In Vivo Studies‐Administration of AAV Vectors

Four‐week‐old male mice were randomly divided into control and PTG overexpression groups. Before the surgical procedures, the mice were anesthetized with Zoletil (at a dose of 0.1 mL per 10 g body weight) and Atropine (at a dose of 0.02 mL per 10 g body weight). Subsequently, the hair around the iWAT of the mice was removed using depilatory cream, and an incision was made in a longitudinal direction on the skin of this area, and the fat pad was exposed using forceps [64]. In each fat pad, multiple sites (four sites per fat pad) were injected with an adeno‐associated virus 9 (AAV9) vector with the adiponectin promoter (WZ Biosciences Inc., Shandong, China). PTG overexpression mice were injected with AAV9‐PTG vector, while the control mice were injected with AAV9‐GFP vector (expressing green fluorescent protein as a control). The volume of injection was 40 µL for each mouse, and the virus titer was maintained at 1 × 10^13^ viral genomes per milliliter (VG/mL). After injection, all mice were kept in a filtered air environment and observed for 4 weeks until stable virus expression.

### 4.3 In Vivo Studies‐PM_2.5_ Concentration Measurement and Component Analysis

PM_2.5_ concentrations were assessed throughout the exposure phase by collecting samples onto Teflon membranes (Whatman, Buckinghamshire, England), which were then subjected to weighing on a Mettler‐Toledo Excellence Plus XP microbalance (Mettler‐Toledo, Greifensee, Switzerland) within a controlled environment that maintained consistent temperature and humidity levels. According to an exposure procedure that required 12 h of exposure per day, the recorded PM_2.5_ concentration was used to calculate the associated average PM_2.5_ concentration. The formula is as follows: the average PM_2.5_ concentration = [12 h × daily PM_2.5_ concentration in the PM_2.5_ chamber (µg/m^3^) + 12 h × daily PM_2.5_ concentration in the FA chamber (µg/m^3^)]/24 h.

The contents of trace metal elements and water‐soluble inorganic ions in PM_2.5_ samples were detected by inductively coupled plasma mass spectrometry and ion chromatography.

### 4.4 In Vivo Studies‐Body Composition

The body composition of mice was measured using a minispec LF50 (Bruker, Germany) after PM_2.5_ exposure. As described previously [32], each mouse was measured twice, separated by a 10 min pause, for accurate recording.

### 4.5 In Vivo Studies‐Glucose Tolerance Test and Insulin Sensitivity Test

After PM_2.5_ exposure, GTT and ITT were conducted. Mice were given a 12 h fast the night before the GTT test. A FreeStyle Blood Glucose Meter (Abbott Diabetes Care Inc., Alameda, CA, USA) was used to measure blood glucose levels at baseline and then 15, 30, 60, 90, and 120 min after receiving an intraperitoneal injection of dextrose (2 mg/g body weight). In the ITT, mice were given an intraperitoneal injection of insulin (0.5 U/kg) after a 4.5 h fast, and blood glucose measurements were made at the same intervals as in the GTT.

### 4.6 In Vivo Studies‐Energy Expenditure Measurement

After 24 h of acclimation, VO_2_ consumption, VCO_2_ production, and heat production were measured at the end of PM_2.5_ exposure using the Columbus Instruments Laboratory Animal Monitoring System (CLAMS) (Columbus, OH, USA) over 24 h.

### 4.7 In Vivo Studies‐Thermal Imaging

Thermal imaging was conducted using a FLIR infrared camera to acquire photographs. These images were then processed through FLIR software to accurately demarcate the inguinal fat region in the mouse. Within the defined area, the software identified the top 10% of temperature points, and the mean temperature value was calculated from this subset to represent the average temperature of the inguinal fat area [65].

### 4.8 In Vivo Studies‐Sample Collection

After PM_2.5_ exposure, mice were immediately euthanized. The iWAT was quickly collected and weighed. Subsequently, a bulk of the iWAT samples from each mouse were preserved in 4% paraformaldehyde for histological examination, while the rest were frozen at −80°C for future use.

### 4.9 In Vivo Studies‐Histological Analysis

For H&E staining, paraffin‐embedded tissue samples were cut into 5 µm sections and then stained with hematoxylin and eosin as described previously [32]. The adipocyte size in iWAT sections was analyzed with ImageJ Fiji 2.1.0 (USA).

For PAS staining, paraffin‐embedded tissue samples were cut into 5 µm sections and then stained with periodic acid and Schiff. The integrated optical density (IOD) of iWAT sections was analyzed with ImageJ Fiji 2.1.0 (USA).

For immunohistochemistry staining, paraffin‐embedded sections (5 µm) underwent deparaffinization, rehydration, and antigen retrieval. Following endogenous peroxidase and non‐specific staining blockage (MXB, Fuzhou, China), sections were incubated with primary antibody (UCP1, Cat# 23673‐1‐AP, Proteintech) overnight at 4°C. Post‐PBS washing, sections were treated with HRP‐conjugated goat anti‐rabbit IgG and streptavidin‐peroxidase (MXB, Fuzhou, China), followed by 3,3′‐diaminobenzidine (DAB) staining (ZSGB, Beijing, China) for visualization. The UCP1 staining positive areas were analyzed by calculating the averaged values of IOD using ImageJ Fiji 2.1.0 (USA).

### 4.10 In Vivo Studies‐Transmission Electron Microscope

The iWAT was prefixed in 2.5% glutaraldehyde in 0.1 M phosphate buffer (pH 7.0) for ≥4 h, followed by triple washing in the same buffer. Post fixation was performed with 1% OsO4 in phosphate buffer for 1–2 h, with subsequent triple washing in the buffer. Serial dehydration was achieved using a gradient of ethanol, followed by a gradient of acetone, and finally in absolute acetone. The specimen was then infiltrated with a 1:1 mixture of absolute acetone and Spurr resin for 1 h at room temperature, followed by a 1:3 mixture for 3 h, and immersed in the final Spurr resin overnight. Polymerization was completed by placing the specimen in an Eppendorf tube containing Spurr resin and heating at 70°C for ≥ 9 h. Sections were cut to 60–80 nm using a LEICA EM UC7 ultramicrotome, stained with uranyl acetate and alkaline lead citrate, and observed under a Hitachi Model HT‐7820 TEM.

### 4.11 In Vivo Studies‐Glycogen Content Measurement

Glycogen Content Assay Kit (BC0345, Solarbio) was used to determine the content of glycogen in iWAT by comparing it to a glycogen standard curve.

### 4.12 In Vivo Studies‐Candidate Gene Selection for Validation

Focusing on PTG (encoded by *Ppp1r3c*) and UCP1 as the core molecules of interest, candidate genes for experimental validation were selected from the GeneMANIA network (http://genemania.org) using a filtering approach. Redundant isoforms (other family members) were first excluded. Then, genes with established roles in adipose metabolism or thermogenesis were screened based on the literature for further verification.

### 4.13 In Vitro Studies‐Cell Culture and Conditional Medium Collection From PM_2.5_‐stimulated Macrophages

To isolate the autonomous cellular effects, the pre‐adipocyte cell line 3T3‐L1 was cultured in Dulbecco's Modified Eagle's Medium (DMEM) (Thermo Fisher Scientific Inc., MA, USA) containing 10% fetal bovine serum (Thermo Fisher Scientific Inc., MA, USA) and penicillin/streptomycin (Absin, Shanghai, China). The cells were maintained at 37°C in an incubator with 5% CO_2_. To induce adipocyte differentiation, the cells were treated with an induction medium including 50 nm insulin (Sigma Aldrich, USA), 100 nm 3,3ʹ,5‐Triiodo‐l‐thyronine (Sigma Aldrich, USA), 0.125 mm indomethacin (Sigma Aldrich, USA), 2 µg/mL dexamethasone (Sigma Aldrich, USA), 0.5 mm isobutylmethylxanthine (Sigma Aldrich, USA). Then the cells were switched to the maintenance medium containing 50 nm insulin, 1 nM 3,3ʹ,5‐Triiodo‐l‐thyronine at day 2 and day 4. 3T3‐L1 adipocytes were matured at day 6 for further experiments.

As previously documented [66], a freshly prepared PM_2.5_ suspension (SRM1648a, NIST) was diluted to a concentration of 10 mg/mL in DMEM culture medium, followed by sonication for a duration of 1 h at room temperature. Subsequently, RAW264.7 cells were treated with PM_2.5_ suspensions at either 25 or 100 µg/mL. Following a 24 h exposure period, the cell culture supernatants were harvested as conditional media and subsequently filtered through 0.22 µm filters. These filtered supernatants were then preserved at a temperature of −80°C for subsequent analyses and experiments.

### 4.14 In Vitro Studies‐PM_2.5_ and Glycogen Treatment

Differentiated 3T3‐L1 adipocytes were treated with different concentrations (0, 25, and 100 µg/mL) of PM_2.5_ conditional medium for 48 h.

Differentiated 3T3‐L1 adipocytes were exposed to varying concentrations (0, 1.25, and 2.5 mg/mL) of glycogen (MedChemExpress, NJ, USA) for 48 h.

### 4.15 In Vitro Studies‐Adenovirus and siRNA Transfection

The cells were transfected with adenovirus (WZ Biosciences Inc., Shandong, China) for 48 h to overexpress PTG and ADRB3, following the manufacturer's protocol.

siRNA was designed and synthesized by GenePharma (Shanghai, China). siRNA was transfected into differentiated adipocytes using Lipofectamine2000 Transfection Reagent (GenePharma, Shanghai, China) for 48 h to knock down PTG and VEGFB, following the manufacturer's protocol.

### 4.16 In Vitro Studies‐Mitochondrial Oxygen Consumption Rate Measurement

Differentiated 3T3‐L1 adipocytes were added to Oroboros O2K. After the respiratory values were stabilized, oligomycin was added to obtain the leak of complex I. Subsequently, carbonyl cyanide m‐chlorophenylhydrazone (CCCP) was added to obtain the maximum electron transfer capacity values, and rotenone/actinomycin A were then sequentially added into the instruments, followed by the measurement of mitochondrial oxygen consumption rate.

### 4.17 In Vivo and In Vitro Studies‐RNA Extraction and Real‐time qPCR

iWAT tissue and 3T3‐L1 adipocytes samples were processed to extract total RNA using the Trizol reagent (TaKaRa, Shiga, Japan), followed by the conversion of RNA to cDNA with the PrimeScript RT Master Mix (TaKaRa, Shiga, Japan). The resultant cDNA served as a template for preparing the reaction mixtures for quantitative polymerase chain reaction (qPCR), which were composed of specific primers (Table S1) and PowerUP SYBR Green Master Mix (Applied Biosystems, CA, USA). Real‐time qPCR was executed on the QuantStudio Q7 Real‐Time PCR system (Applied Biosystems, CA, USA). The relative mRNA expression levels were quantified using the ΔΔCT method and were standardized to the expression of *β‐actin*.

### 4.18 In Vivo and In Vitro Studies‐Western Blotting

A protease inhibitor combination was added to the RIPA lysis solution (Boster Biological Technology Co., Ltd., California, USA) in order to separate the iWAT tissue and 3T3‐L1 adipocytes' proteins. Following portioning, protein lysates were separated on 10% SDS‐PAGE gels. Following resolution, the proteins were blotted onto PVDF membranes and blocked using either skim milk or 5% bovine serum albumin (BSA). The primary antibodies (Table S2) were incubated with the immunoblots for a whole night at 4°C. The membranes were then washed and incubated with a horseradish peroxidase‐conjugated secondary antibody. Lastly, the ChemiDoc Imaging System (Bio‐Rad, Hercules, California, USA) was used to view the target proteins, and ImageJ Fiji 2.1.0 (USA) was used to quantify them.

### 4.19 Statistical Analysis

Replicates are described in the figure legends. All data are shown as means ± standard error of the mean (SEM). All statistical calculations and graphs were created using GraphPad Prism Ver 9.5.0. When applicable, Student's *t*‐test, One‐Way analysis of variance (ANOVA) followed by Tukey's test, and Two‐way ANOVA with Tukey's multiple comparison test were used to evaluate the statistical significance. Correlations were examined with the non‐parametric Spearman correlation test. Statistical significance was set at *P* < 0.05.

## Author Contributions

C.L. conceived and designed the study and supervised all research activities. L.W., R.H., S.L., L.Z., and W.Z. performed animal experiments. L.W., Y.C., and P.H. performed cell culture and in vitro experiments. L.W. performed the histology and imaging analysis. L.Z., L.Q., R.L., X.H., and Q.S. provided technical or material support and consultation. L.W. wrote the original manuscript draft. C.L. critically reviewed, revised, and approved the final version of the manuscript.

## Funding

This work was supported by the National Natural Science Foundation of China (Grant Nos. 82273590, 81973001, 82173480, 82004143), Key Research and Development International Cooperation Projects (2019YFE0114500).

## Ethical Approval

All animal experiments followed the ARRIVE guidelines. Ethical approval was obtained from the Institutional Animal Care and Use Committee (IACUC) of Zhejiang Chinese Medical University (IACUC‐202409‐23).

## Conflicts of Interest

The authors declare no conflicts of interest.

## Supporting information




**Supporting File**: advs73752‐sup‐0001‐SuppMat.docx.

## Data Availability

The data that support the findings of this study are available from the corresponding author upon reasonable request.
