# Supplementary material for: PTG‐Dependent Glycogen Metabolic Dysfunction Drives Impaired Adipose Browning: A Novel Mechanism Linking PM2.5 to Metabolic Disorders
Source: Adv Sci (Weinh). 2026 Jan 9;13(16):e12589. doi: 10.1002/advs.202512589 (PMC13042893; doi:10.1002/advs.202512589)
Supplement: Supplementary file 1 — Supporting File: advs73752‐sup‐0001‐SuppMat.docx. [file ADVS-13-e12589-s001.docx]

Supplemental materials for

**PTG-dependent** **Glycogen Metabolic Dysfunction Drives Impaired Adipose Browning: A Novel Mechanism Linking PM_2.5_ to Metabolic Disorders**

Limin Wang^a,b^, Renjie Hu^a,b^, Yanxi Chai^a,b^, Ping He^a,b^, Sanduo Li^a,b^, Lisha Zhao^a,b^, Wenbin Zhao^a,b^, Lu Zhang^a,b^, Li Qin^a,b^, Ran Li^a,b^, Xiaoli Hou^c^, Qinghua Sun^a,b^, Cuiqing Liu^a,b*^

^a^ School of Public Health, Zhejiang Chinese Medical University, Hangzhou, Zhejiang, China

^b^ Zhejiang International Science and Technology Cooperation Base of Air Pollution and Health, Hangzhou, Zhejiang, China

^c^ Academy of Chinese Medical Sciences, Zhejiang Chinese Medical University, Hangzhou, Zhejiang, China

^*^Correspondence:

Cuiqing Liu, Ph.D.

liucuiqing@zcmu.edu.cn

School of Public Health, Zhejiang Chinese Medical University, Zhejiang International Science and Technology Cooperation Base of Air Pollution and Health

Hangzhou 310053

China

Additional file: Table S1-2 and Figure S1-10

**Table S1.** Primers used for quantitative RT-PCR.

| **Genes** | **Forward primer** | **Reverse primer** |
| --- | --- | --- |
| *Acly* | ACCCTTTCACTGGGGATCACA | GACAGGGATCAGGTATTCCTTG |
| *Angptl3* | TGCACCTTCAGAGCCAAAAT | CATTGGTTCGAAGTGATAGGTCA |
| *Atp5a1* | CGGGACTGGTCTCCAAAAATG | CGTGTCAGCTCCCAGAATCC |
| *Atp5k* | TCAAGTTCGGCCGGTACTC | CCGCTGCTATTCTTCTCTCCT |
| *CD137* | CCAAGTACCTTCTCCAGCATAGG | GCGTTGTGGGTAGAGGAGCAAA |
| *Cidea* | GGTGGACACAGAGGAGTTCTTTC | CGAAGGTGACTCTGGCTATTCC |
| *Cox5a* | CAACCCGCGAGCTTACA | GTATATTGTGTCCTCGGAC |
| *Cox6a1* | CGAGAGACCCCCGTTCGT | GTTGTGGAAGAGGGTATGGTTACC |
| *Cox8b* | GAACCATGAAGCCAACGACT | GCGAAGTTCACAGTGGTTCC |
| *Dio2* | GGTGGTCAACTTTGGTTCAGCC | AAGTCAGCCACCGAGGAGAACT |
| *Gys1* | ATCTACACTGTGCTGCAGACG | CCCTTGCTGTTCATGGAATCC |
| *Gys2* | CCATCCTCAGCACCATTAGAC | GTGACAACCTCGGACAAACTC |
| *Lpl* | AGAGCCAAAAGAAGCAG | GGCAGAGTGAATGGGAT |
| *Lrp1* | GGACCACCATCGTGGAAA | TCCCAGCCACGGTGATAG |
| *Mdh1* | TAAGGTTATCGTGGTGGG | TGCTTTAGCTCGGTTGTG |
| *Ndufa1* | CAGGCCCTTGGACACATAGT | GTCCACTGCGTACATCCACA |
| *Ndufb9* | TCCAAGAGAGAGCAGTGGAAGAA | AGGAGGCAAAGCTTCAGTCATAA |
| *Pgc1α* | AGCCGTGACCACTGACAACGA | GCTGCATGGTTCTGAGTGCT |
| *Ppp1r3c* | TGATCCATGTGCTAGATCCACG | ACTCTGCGATTTGGCTTCCTG |
| *Pygl* | TGCTTTGGATAAGAAGGGGTATGAGGC | TTGAAGAGGTCTGGCTGATTGGGAG |
| *Sdhb* | TGGATCTGAATAAGTGCGGACC | GCCAGAGTATTGCCTCCGTT |
| *Ucp1* | GAGGTCGTGAAGGTCAGAAT | CTGTGGTGGCTATAACTCTGTAA |
| *Uqcrb* | AGGCTTCCTGAGGACCTTTA | TCCTTAGGCAAGATCTGATGC |
| *Vegfa* | GGAGATCCTTCGAGGAGCACTT | GGCGATTTAGCAGCAGATATAAGAA |
| *Vegfb* | TCTGAGCATGGAACTCATGG | TCTGCATTCACATTGGCTGT |
| *Vegfc* | GGGGGCGAGGTCAAGGCTTTT | CCTGGTATTGAGGGTGGGCTGC |
| *β-actin* | TGTGATGGTGGGAATGGGTCAGAA | TGTGGTGCCAGATCTTCTCCATGT |

**Table S2.** Primary antibodies used for immunoblotting

| **Antibody** | **Dilutions** | **Catalogue #** | **Company** |  |
| --- | --- | --- | --- | --- |
| ADRB3 | 1:1000 | Cat# ab94506 | Abcam |  |
| UCP1 | 1:1000 | Cat# 23673-1-AP | Proteintech |  |
| PTG | 1:1000 | Cat# 29424-1-AP | Proteintech |  |
| NDUFB9 | 1:500 | Cat# HA721015 | HUABIO |  |
| SDHA | 1:1000 | Cat# A2594 | ABclonal |  |
| UQCRFS1 | 1:1000 | Cat# A6784 | ABclonal |  |
| COX5A | 1:1000 | Cat# A6437 | ABclonal |  |
| ATPB | 1:1000 | Cat# A5769 | ABclonal |  |
| VEGFB | 1:1000 | Cat# A2132 | ABclonal |  |
| hGS | 1:1000 | Cat# 22371-1-AP | Proteintech |  |
| hGP | 1:1000 | Cat# 15851-1-AP | Proteintech |  |
| mGS | 1:1000 | Cat# 10566-1-AP | Proteintech |  |
| HSP90 | 1:5000 | Cat# 13171-1-AP | Proteintech |  |


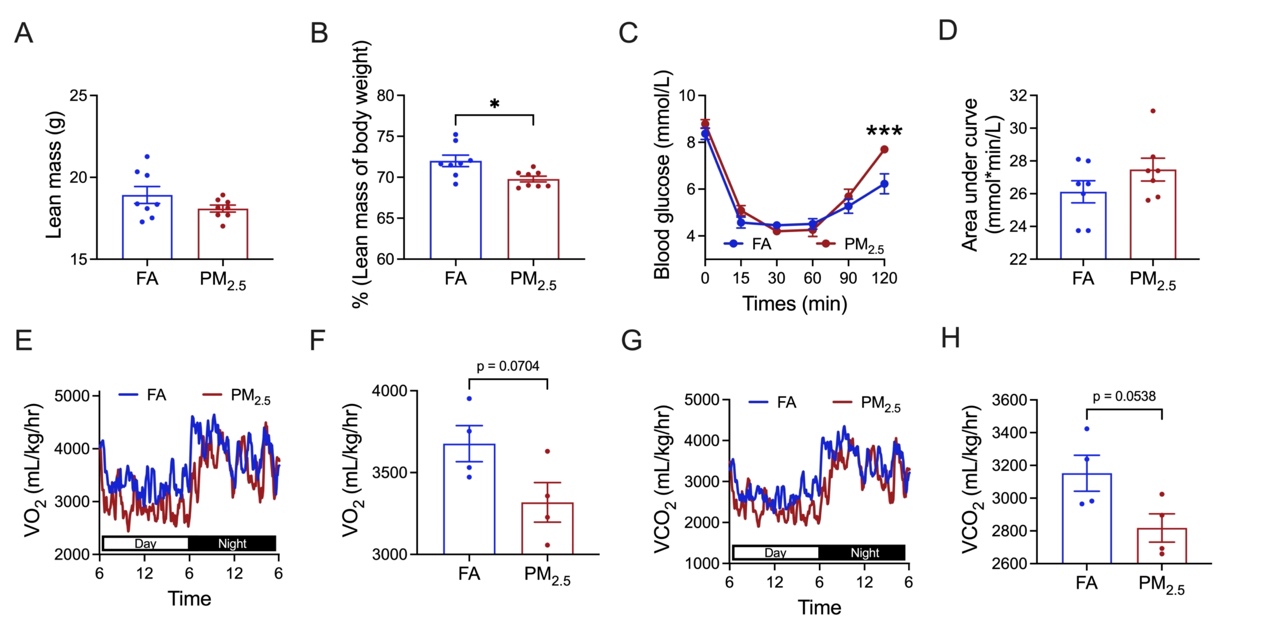


**Fig. S1.** Effects of PM_2.5_ exposure on lean mass, the ratio of lean mass to body weight, insulin sensitivity, oxygen consumption, and carbon dioxide release in mice. **A-B**. lean mass (**A**) and the ratio of lean mass to body weight (**B**) of mice (*n* = 8 per group). **C-D**. ITT (**C**) and AUC of ITT (**D**) of mice (*n* = 7 per group). **E-F**. Oxygen consumption of mice (*n* = 4 per group). Line graph (**E**) indicates variation patterns of oxygen consumption of mice over a 24h period, column graph (**F**) indicates the mean values of oxygen consumption of mice over a 24h period. **G-H**. Carbon dioxide release of mice (*n* = 4 per group). Line graph (**G**) indicates variation patterns of carbon dioxide release of mice over a 24h period, column graph (**H**) indicates the mean values of carbon dioxide release of mice over a 24h period. Data were presented as mean ± SEM, analyzed by unpaired, two-tailed Student’s t test (A, B, D, F, and H) or two-way ANOVA with Tukey’s multiple comparisons test (C). **P* < 0.05, ****P* < 0.001.


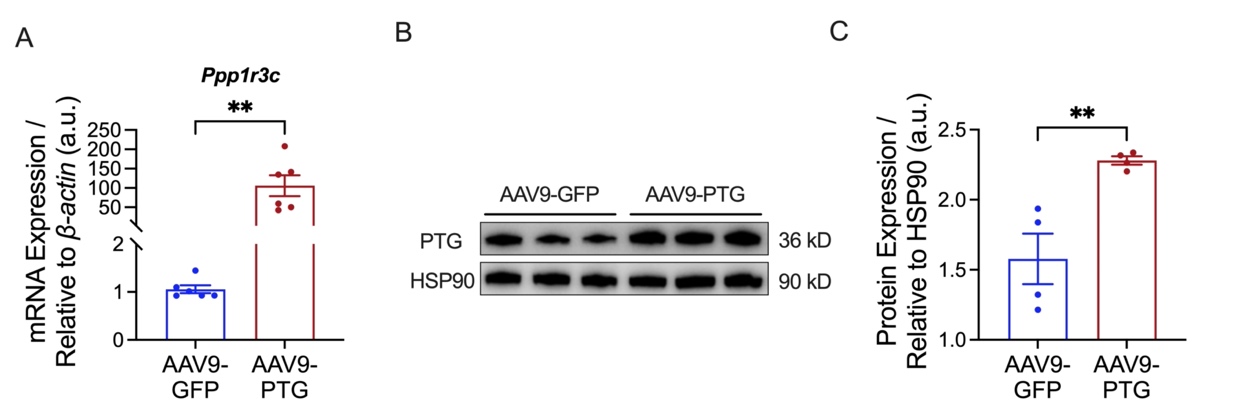


**Fig. S2.** Construction and efficiency verification of specific PTG overexpression mouse model. **A.** mRNA expression of *Ppp1r3c* in iWAT (*n* = 6 per group). **B-C**. Representative bands (**B**) and quantitative analysis (**C**) of PTG in iWAT (*n* = 4 per group). Data were presented as mean ± SEM, analyzed by unpaired, two-tailed Student’s t test. ***P* < 0.01.


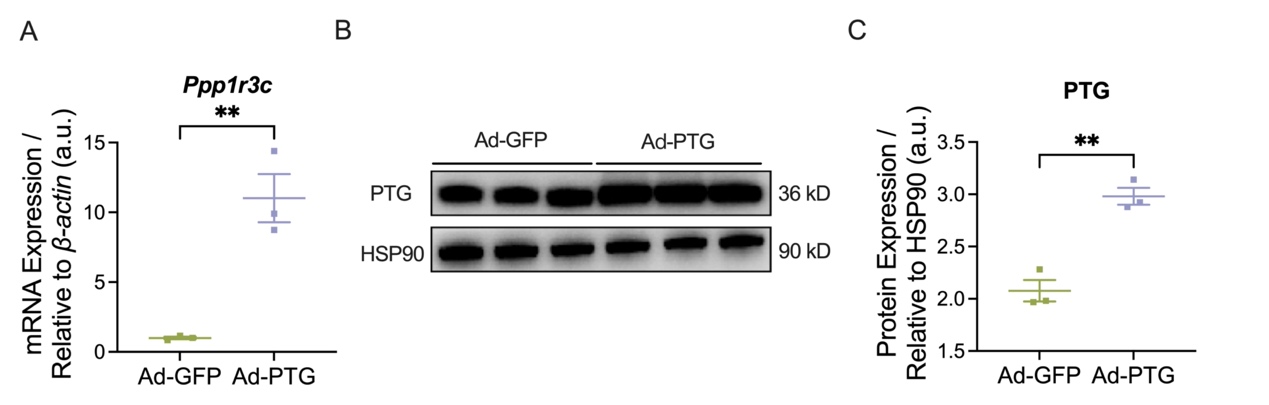


**Fig. S3.** Construction and efficiency verification of PTG overexpression cell model. **A.** mRNA expression of *Ppp1r3c* in 3T3-L1 adipocytes (*n* = 3 per group). **B-C**. Representative bands (**B**) and quantitative analysis (**C**) of PTG in 3T3-L1 adipocytes (*n* = 3 per group). Data were presented as mean ± SEM, analyzed by unpaired, two-tailed Student’s t test. ***P* < 0.01.


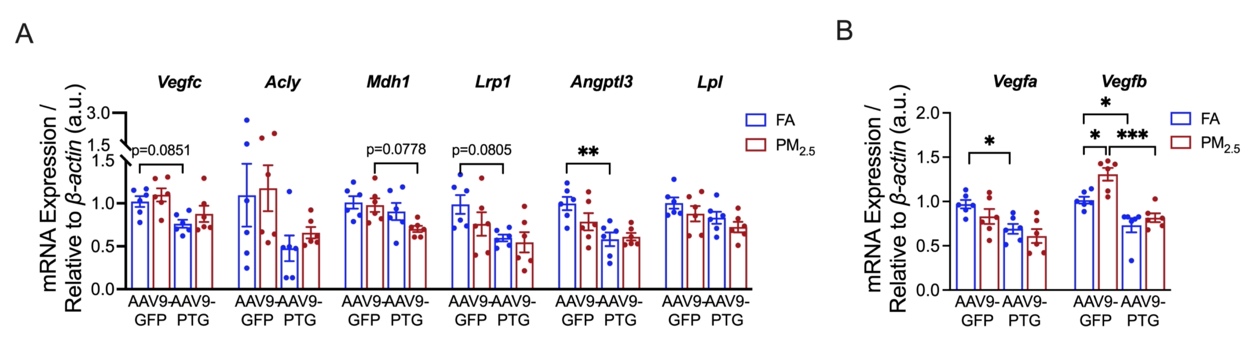


**Fig. S4.** Validation of molecular targets. **A**. mRNA expression of *Vegfc, Acly, Mdh1, Lrp1, Angptl3,* and *Lpl* in 3T3-L1 adipocytes (*n* = 3 per group). **B**. mRNA expression of *Vegfa* and *Vegfb* in 3T3-L1 adipocytes (*n* = 3 per group). Data were presented as mean ± SEM, analyzed by two-way ANOVA with Tukey’s multiple comparisons test. **P* < 0.05, ***P* < 0.01, ****P* < 0.001.


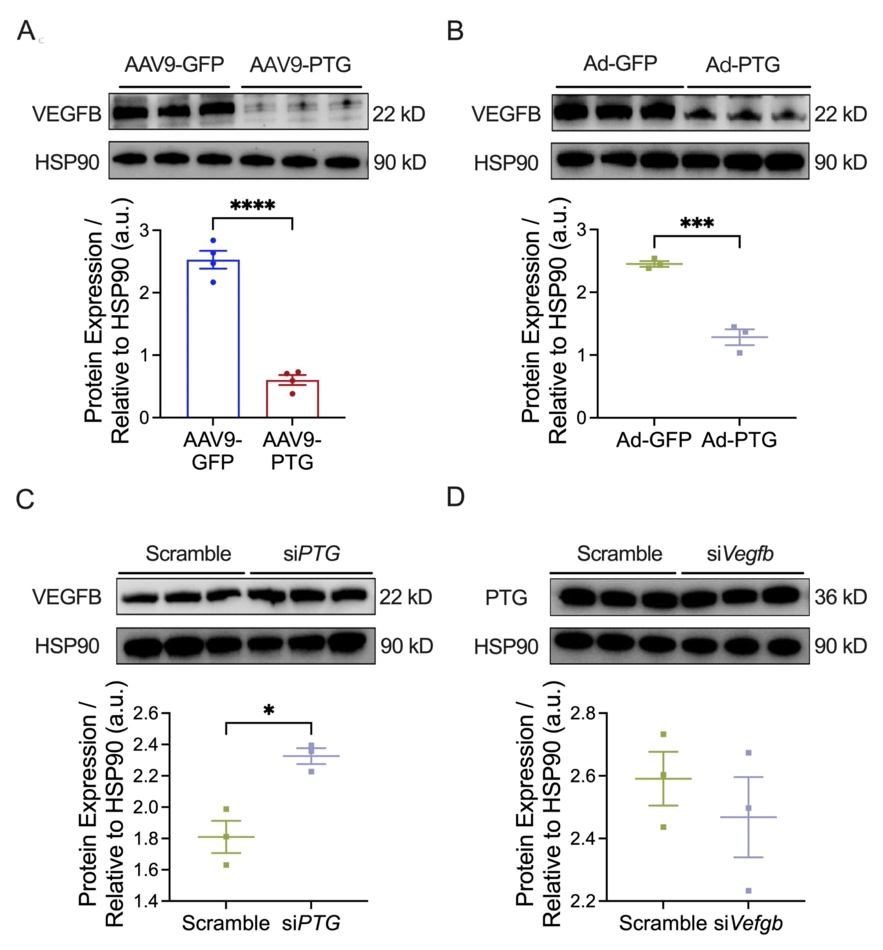


**Fig. S5.** Regulation of VEGFB by PTG. **A**. Representative bands (**top**) and quantitative analysis (**bottom**) of VEGFB in iWAT (*n* = 4 per group). **B**. Representative bands (**top**) and quantitative analysis (**bottom**) of VEGFB in 3T3-L1 adipocytes (*n* = 3 per group). **C**. Representative bands (**top**) and quantitative analysis (**bottom**) of VEGFB in 3T3-L1 adipocytes (*n* = 3 per group). **D**. Representative bands (**top**) and quantitative analysis (**bottom**) of PTG in 3T3-L1 adipocytes (*n* = 3 per group). Data were presented as mean ± SEM, analyzed by unpaired, two-tailed Student’s t test. **P* < 0.05, ****P* < 0.001, *****P* < 0.0001.


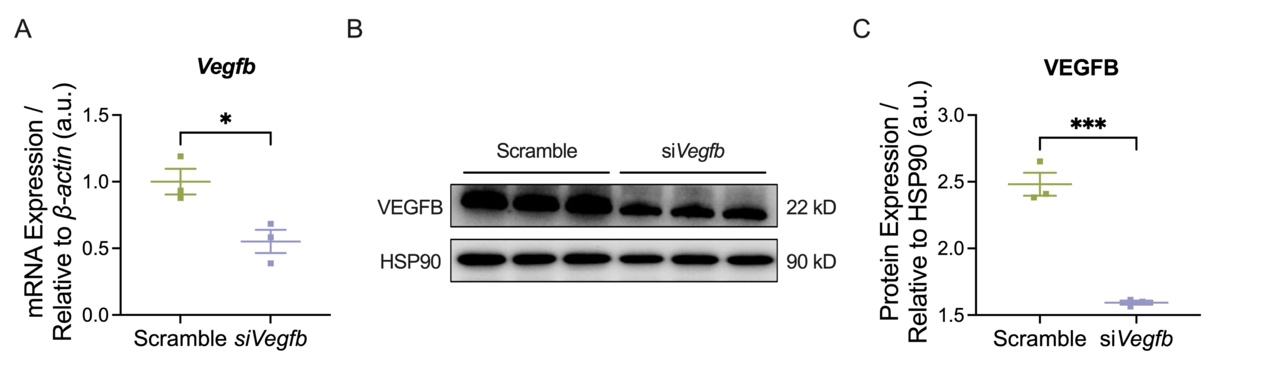


**Fig. S6.** Construction and efficiency verification of VEGFB knockdown cell model. **A.** mRNA expression of *Vegfb* in 3T3-L1 adipocytes (*n* = 3 per group). **B-C**. Representative bands (**B**) and quantitative analysis (**C**) of VEGFB in 3T3-L1 adipocytes (*n* = 3 per group). Data were presented as mean ± SEM, analyzed by unpaired, two-tailed Student’s t test. **P* < 0.05, ****P* < 0.001.


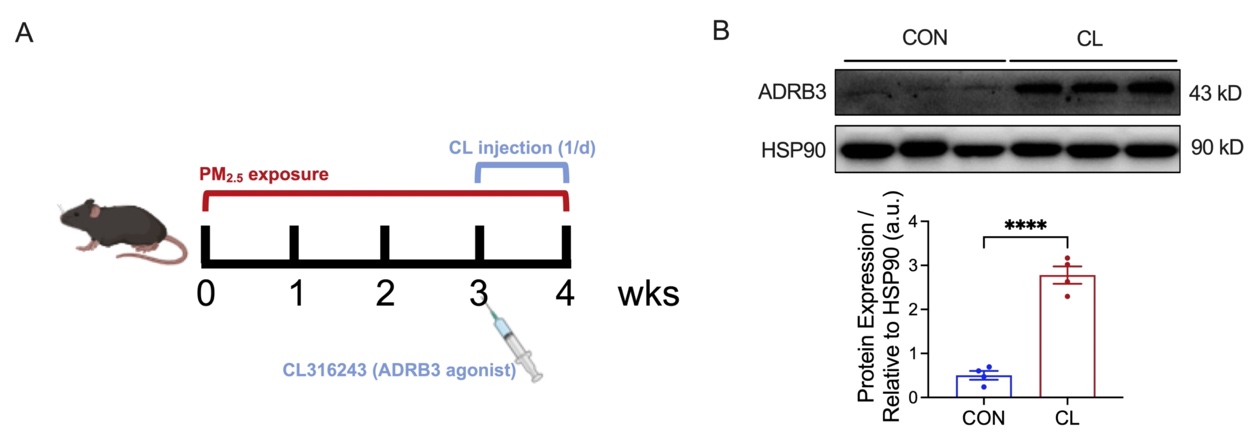


**Fig. S7.** Construction and efficiency verification of ADRB3 activation mouse model. **A.** Schematic representation of CL mice by CL316243 injection. **B-C**. Representative bands (**top**) and quantitative analysis (**bottom**) of ADRB3 in iWAT (*n* = 4 per group). Data were presented as mean ± SEM, analyzed by unpaired, two-tailed Student’s t test. *****P* < 0.0001.


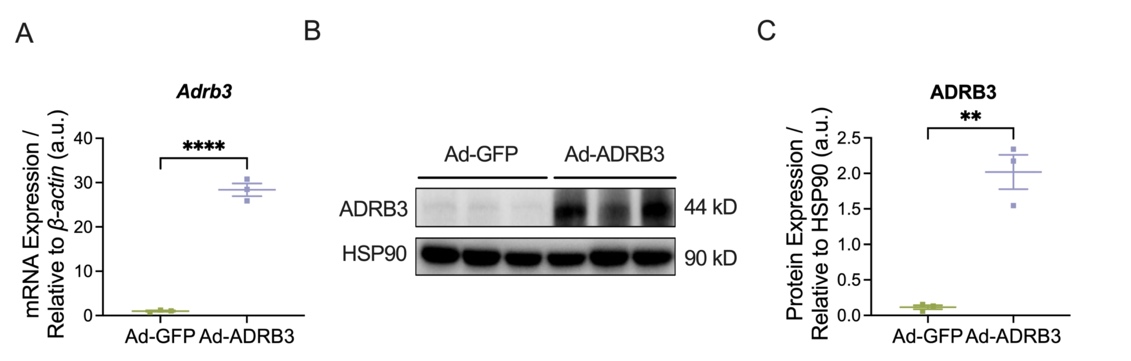


**Fig. S8.** Construction and efficiency verification of ADRB3 overexpression cell model. **A.** mRNA expression of *Adrb3* in 3T3-L1 adipocytes (*n* = 3 per group). **B-C**. Representative bands (**B**) and quantitative analysis (**C**) of ADRB3 in 3T3-L1 adipocytes (*n* = 3 per group). Data were presented as mean ± SEM, analyzed by unpaired, two-tailed Student’s t test. ***P* < 0.01, *****P* < 0.0001.


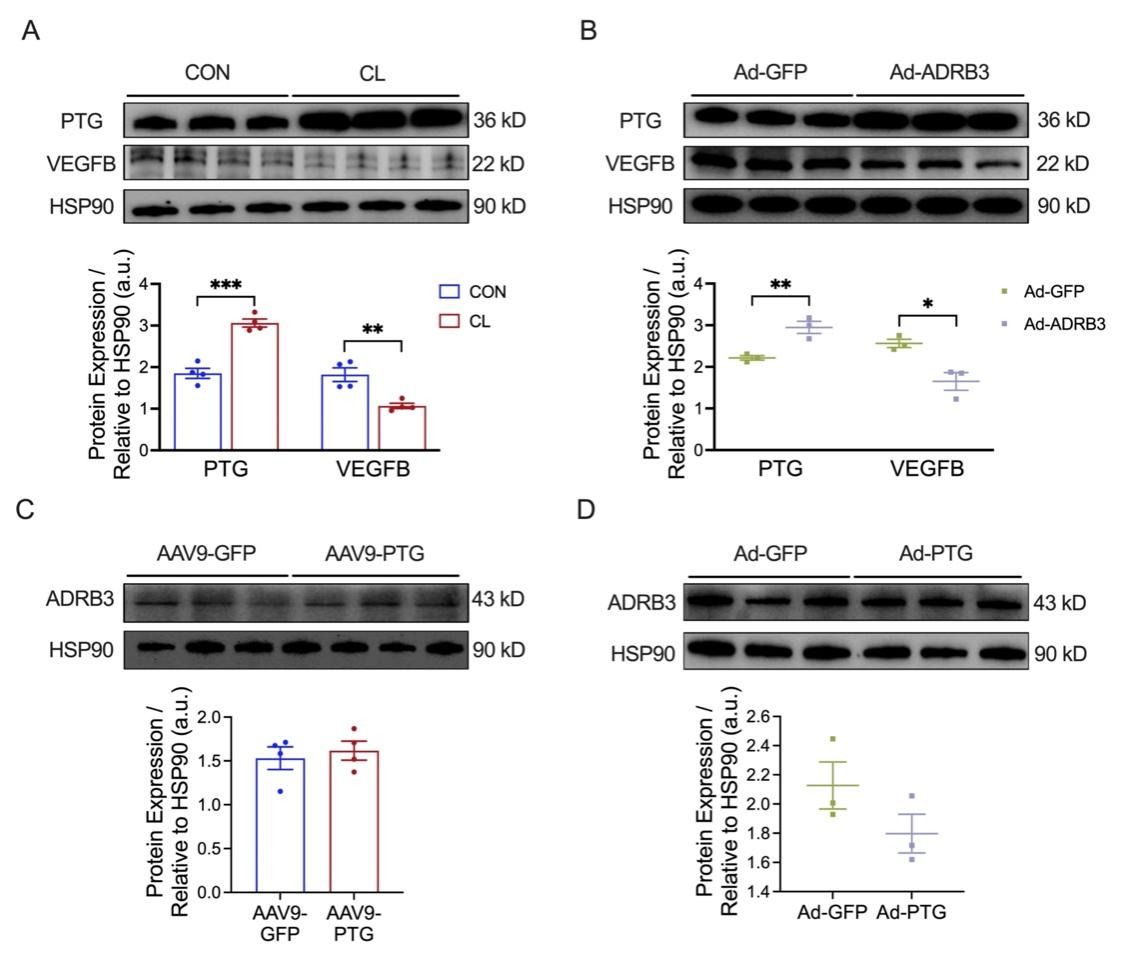


**Fig. S9.** Regulation of PTG and VEGFB by ADRB3. **A**. Representative bands (**top**) and quantitative analysis (**bottom**) of PTG and VEGFB in iWAT (*n* = 4 per group). **B**. Representative bands (**top**) and quantitative analysis (**bottom**) of PTG and VEGFB in 3T3-L1 adipocytes (*n* = 3 per group). **C**. Representative bands (**top**) and quantitative analysis (**bottom**) of ADRB3 in iWAT (*n* = 4 per group). **D**. Representative bands (**top**) and quantitative analysis (**bottom**) of ADRB3 in 3T3-L1 adipocytes (*n* = 3 per group). Data were presented as mean ± SEM, analyzed by unpaired, two-tailed Student’s t test. **P* < 0.05, ***P* < 0.01, ****P* < 0.001.


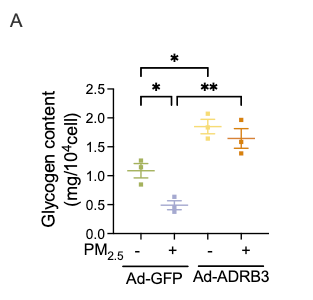


**Fig. S10.** Effects of ADRB3 overexpression on glycogen content in 3T3-L1 adipocytes. **A.** Glycogen content in 3T3-L1 adipocytes (*n* = 3 per group). Data were presented as mean ± SEM, analyzed by two-way ANOVA with Tukey’s multiple comparisons test. **P* < 0.05, ***P* < 0.01.
